# Aging-related behavioral defects in *Caenorhabditis elegans*

**DOI:** 10.1242/bio.062711

**Published:** 2026-08-17

**Authors:** Jingxuan Zeng, Chung-Kuan Chen, Yuichi Iino, Yu Toyoshima

**Affiliations:** ^1^ Department of Biological Sciences, Graduate School of Science, University of Tokyo, Tokyo 113-0031, Japan; ^2^ Department of Computational Biology and Medical Sciences, Graduate School of Frontier Sciences, University of Tokyo, Chiba 277-8562, Japan

**Keywords:** *Caenorhabditis elegans*, Aging, Locomotion

## Abstract

With the rapid development of quantitative behavioral analysis, locomotion behaviors can be examined and described in greater detail. However, behavioral structures underlying the dysfunctions and locomotor defects observed with aging remain poorly understood. Here, we recorded and analyzed worms’ locomotion movement for both young and aged individuals. The results revealed distinct locomotion patterns between the two groups. Compared to the young worms, the aged individuals exhibited more disorganized forward locomotion, characterized by reduced periodicity and lower cycle stability. The time duration and frequency of backward locomotion did not differ significantly between the two groups, whereas turn behavior following reversals was significantly reduced. Moreover, the aged worms showed increased individual heterogeneity in the usage of behavioral repertoire with age. In summary, this study systematically characterizes the behavior defects of aged animals by quantifying changes in locomotor states, event transitions, and behavior organization.

## Teaser

**Summary:** We developed a quantitative framework to analyze age-associated locomotor defects in *Caenorhabditis elegans*, revealing reduced stability of forward movement and altered transitions between behavioral states in aged worms.

## INTRODUCTION

Natural aging is an internal process accompanied by a progressive functional decline and a high risk of neurodegenerative diseases such as Parkinson's disease and Alzheimer's disease in humans. Mitochondrial dysfunction, disordered energy metabolism, misregulated DNA duplication, inflammation, detectable changes in fine ultrastructural regions in the brain and other cellular and molecular changes have been well studied in recent years (Koch et al., 2021; Naseer et al., 2021; Soo et al., 2023). On the other hand, changes in behavioral patterns during aging have attracted much attention (Vaillancourt and Newell, 2002). Although there are huge differences in evolution and a large span of life cycle among species, the mechanisms of such changes might be preserved across a wide range of species, including *Caenorhabditis elegans*.

*C. elegans* has multiple advantages in studying aging, including a short life cycle (Muschiol et al., 2009; Zhang et al., 2020), transparent body, clean genetic background, and sophisticated genetics, making it an ideal model organism (Shen et al., 2018). In *C. elegans*, aspects of functional declines that occur with aging have been studied. For example, some morphological changes in synaptic ultrastructure, decreased number of vesicles and soma volume, and neurite branching and beading were observed in some neurons (Hess et al., 2019; Pan et al., 2011; Toth et al., 2012), which may be related to decreased touch sensitivity in aged animals. Moreover, defects in thermotaxis, chemotaxis and long-term associative learning have also been recognized (Huang et al., 2020; Kauffman et al., 2010). Many studies have also leveraged *C. elegans* to screen candidate therapeutic targets and/or reasons for neurodegenerative diseases such as Alzheimer's disease (Gowda et al., 2026; Li et al., 2019; Pahal et al., 2025; Ros and Carrascosa, 2020) and to investigate how dietary and reactive oxygen species influence lifespan and aging-related phenotypes (Gusarov et al., 2021; Ros and Carrascosa, 2020).

In the aspect of locomotion, it is well established that *C. elegans* exhibits three basic locomotion behaviors: forward movement, backward movement, and turn. This simple behavioral repertoire has enabled substantial progress in the quantitative characterization of behavior in young adult worms. For example, Ahamed et al. (2021) identified a low-dimensional space dominated by three groups of cyclic trajectories corresponding to the worm’s forward, backward and turn. Another study defined stereotyped posture dynamics as discrete behaviors and projected the time point of an animal's posture dynamics onto a two-dimensional map to investigate the behavior state transitions (Liu et al., 2018). By contrast, studies of locomotion in aged animals mainly focused on basic description. A progressive decay in locomotion in early life was observed in the 1970s. Defects such as a decreased motion speed (Hahm et al., 2015) and forward duration and an increased frequency of reversal were defined (Glenn et al., 2004; Liu et al., 2013; Wirak et al., 2022), which may not be due to the decline in body-wall muscles but rather the functional decline in the nervous system (Liu et al., 2013). However, compared with the sophisticated frameworks established in young adult studies (Ahamed et al., 2021; Broekmans et al., 2016; Chung et al., 2024; Costa et al., 2024; Denham et al., 2018; Johari et al., 2013; Liu et al., 2018; Peliti et al., 2013; Roberts et al., 2016; Stephens et al., 2010), a comprehensive quantitative characterization of locomotion in aged animals is still lacking.

To bridge this gap, we recorded and analyzed worms’ locomotion movement for both young and aged individuals and revealed distinct locomotion patterns between the two groups. Young worms exhibit more pronounced and repetitive cyclical patterns, forming tighter loops and clear periodicity. In contrast, aged worms show fewer distinct cycles and more irregular, seemingly chaotic trajectories. Moreover, the aged worms showed increased individual heterogeneity in the usage of behavioral repertoire with age.

## RESULTS

### Aged animals have decreased forward-locomotion frequency and amplitudes

**Fig. 1.**
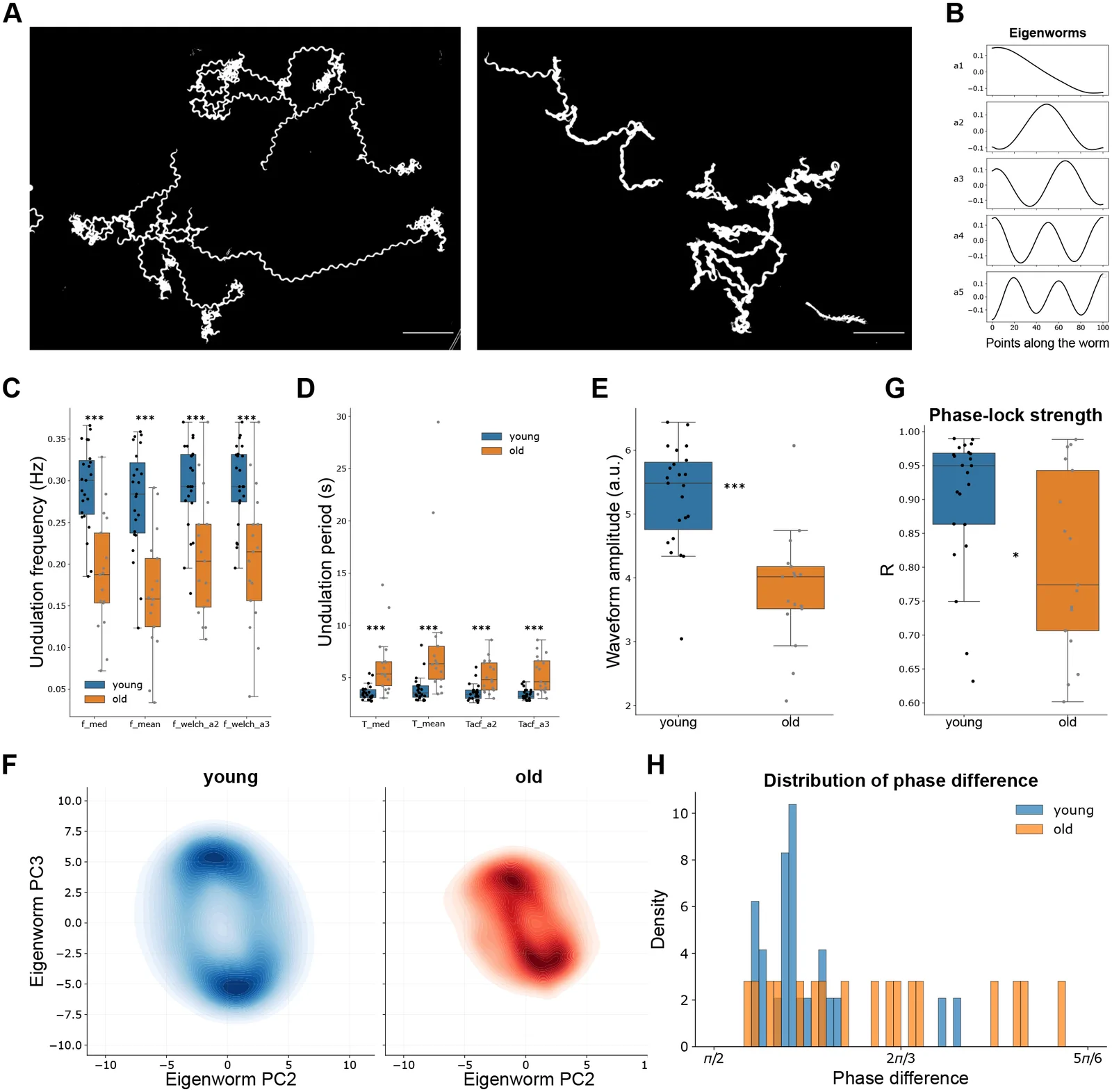
**Forward-locomotion differences.** (A) 5-min locomotion trajectories. Locomotion trajectories were recorded for 5 min from six individuals in the young group (left) and seven individuals in the aged group (right). Scale bars: 5 mm. (B) Five eigenworms. The *x*-axis represents the body position, and the *y*-axis represents the amplitude of each eigenworm. (C-E,G) Each dot represents the estimated value from an individual worm, Mann-Whitney *U* test, *P<0.05, ***P<0.001. Boxes indicate the interquartile range, centerlines indicate the median, and whiskers extend to 1.5× the interquartile range. The statistical analysis was performed at the level of individual worms. Sample sizes were *n*=16 worms for the young group and *n*=16 worms for the aged group. (C) Instantaneous undulation frequency is estimated by multiple methods. Phase-based estimates were obtained from the eigenworm phase dynamics and summarized as the median (f_med) or mean (f_mean) values. Dominant undulation frequencies from the peak of the Welch power spectrum of a2 (f_welch_a2) or a3 (f_welch_a3) are also compared. (D) Corresponding undulation periods are estimated by multiple methods. Phase-based period estimates were calculated from the instantaneous undulation frequency and summarized as the median (T_med) or mean (T_mean). Dominant period estimated from the first non-zero-lag peak of autocorrelation of a2 (Tacf_a2) or a3 (Tacf_a3). (E) Amplitude of sinusoidal waveform during forward movement. (F) Eigenworm state-space density. (G) Phase locking between a2 and a3 during forward locomotion. This measures the consistency of the phase relationship between two signals. (H) Histogram of the eigenworm phase offset. This measures the average angle of the phase difference over time.

It is commonly known that *C. elegans* locomotion behavior decreased with age (Glenn et al., 2004; Herndon et al., 2002; Liu et al., 2013). Also, a previous study had demonstrated that 9-day worms showed a disruption of excitatory-inhibitory neural signaling balance (Wirak et al., 2022). Therefore, we used day 9 as an aged group in our experiments and compared them with day 1 adults (young group). To further describe these deficits in the aged animals, we performed locomotion assays in the two groups. Similar to previous studies (Flavell et al., 2020; Wirak et al., 2022), young worms crawled forward with frequent backward and turn movements (known as local search state), while aged worms showed more intermittent locomotion with increased hesitation and pauses (Fig. 1A). Consistent with these differences, the distribution of locomotion speed is different between groups (Fig. S1). Also, aged worms had a higher tendency to stop their movement at the last half of the recording time (see below for details), as previous research has shown (Glenn et al., 2004). Moreover, unlike the typical sinusoidal locomotion observed in the young worms (Movie 1), the anterior body in the aged worms maintained a clear sinusoidal waveform, whereas the posterior body exhibited markedly reduced bending amplitude, appearing flaccid and passively dragged forward (Movie 2). To quantitatively characterize this phenotype, we extracted the worm centerline for each individual using WormTracer (Kuze et al., 2025) and performed principal component analysis to obtain five eigenworms (Fig. 1B) (Stephens et al., 2008), which captured more than 99% of the variability (Fig. S2). The first eigenworm (a1) contributes to a constant curvature throughout the body, and the second and third eigenworms (a2 and a3) are sinusoidally oscillated, while the last two eigenworms contribute most to the postures of the head and the tail (Fig. 1B).

We next quantified forward-movement undulation dynamics by computing the eigenworm phase *θ*(*t*) and then deriving the corresponding instantaneous undulation frequency *f*(*t*) and the corresponding periods *T*(*t*) (1 / *f*(*t*)). To validate these phase-based estimates, we compared them with independent frequency and period measures obtained from autocorrelation-based peak detection and Welch spectral analysis (Fig. 1C,D). These results demonstrated that young worms exhibited a higher frequency of forward sinusoidal movement, partially consistent with the previous study (Glenn et al., 2004). Undulation strength, or the amplitude of sinusoidal waveform, quantified by the eigenworm-plane amplitude *r*(*t*), showed a reduction in the aged animals (Fig. 1E). To characterize the forward locomotion dynamics in the eigenworm space, we also projected trajectories onto the (a2, a3) plane as phase portraits (Fig. 1F), which revealed a stable and broad loop in the young group while a collapsed occupancy in the aged group, indicating an altered posture dynamics and irregular locomotion waveform. Hilbert-based phase analysis revealed a weaker phase locking between a2 and a3 in the aged group compared to the young group, as indicated by a reduced phase-locking strength (Fig. 1G). This indicated that the coordination between a2 and a3 became less consistent over time in the aged group. Additionally, the mean phase offset suggested a more pronounced phase lag in the aged animals, which means that in the aged worms, eigenworm 3 tended to be delayed relative to eigenworm 2 by a larger phase difference. In the young worms, the phase delay was mainly concentrated between π/2 and 2π/3, whereas in the aged worms, the phase-delay distribution was broader and more dispersed (Fig. 1H). These findings are consistent with previous results obtained using a graph-neural-network-based predictive model (Yuan et al., 2023).

### Dimension reduction revealed several locomotion states

**Fig. 2.**
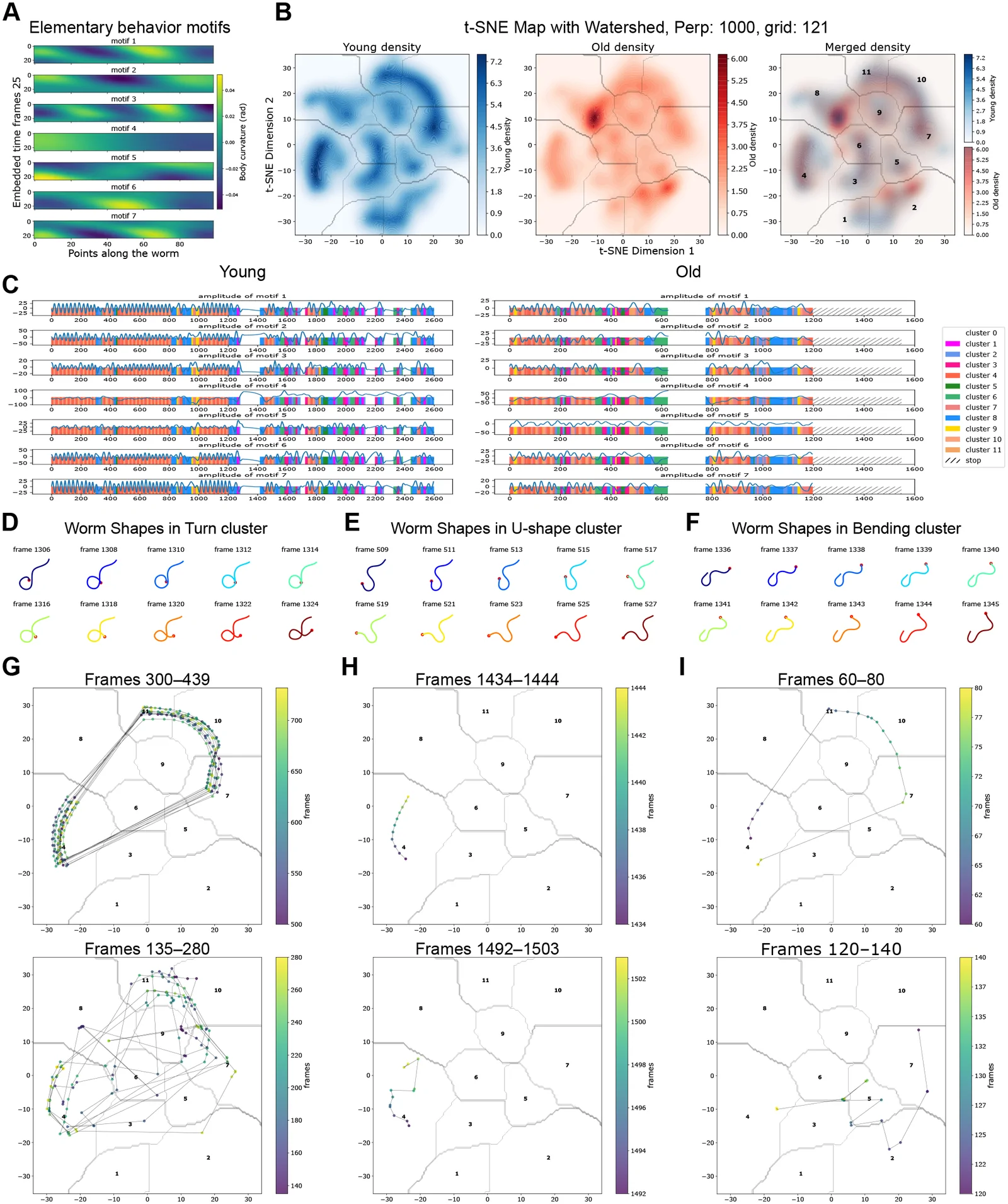
**Locomotion behavior classification based on t-SNE and watershed clustering.** (A) Elementary behavior motifs. The *x*-axis represents the body position, and the *y*-axis represents the 25 embedded frames. (B) t-SNE probability density of behavioral motif vectors. Clusters were segmented by a watershed algorithm. (C) Behavioral motif dynamics along time frames for a young (left) and an aged (right) worm. Colors indicate distinct behavioral clusters. (D-F) Representative worm shapes in the turn, U-shape and bending clusters, respectively. Red dots indicate the worm's head. (G-I) Forward-movement trajectories on t-SNE map. The colored trajectory corresponds to a continuous time-series movement. Each panel shows one representative sample, with the corresponding time frames indicated in the panel title. (G) One sample each from the young (upper) and aged (lower) groups. The samples shown here are the same as those in Movies 1, 2, 7, and 8. (H) Irregular forward locomotor cycles, case 1. Compared with the upper panel (Movie 9), the lower panel (Movie 10) shows nonuniform curvature across consecutive time points. (I) Irregular forward-locomotor cycles, case 2. Compared with the upper panel (Movie 11), consecutive time points in the lower panel (Movie 12) showed more frequent transition into other clusters.

To further investigate *C. elegans* locomotor behavior beyond forward movement, we performed time-delay embedded reconstruction independent component analysis (TDE-RICA) to capture temporal motif patterns instead of instantaneous worm postures (Fig. S3). In this analysis, we first used interpolation to resample the young data so that two groups share the same undulation frequency during forward locomotion (see Materials and Methods, Fig. S4). A previous study had demonstrated that seven independent components could reach a saturation in predictability when the embedding window is set to approximately half of the body wave cycle (Ahamed et al., 2021). Here, we utilized similar embedded dimensions (see Materials and Methods) and obtained seven elementary behavior motifs (Fig. 2A) and corresponding weights (motif occurrences). Unlike the study by Ahamed et al. (2021), our seven behavior motifs fall into only two groups: forward (motifs 1, 2, 3, 6, and 7) and turn behaviors (motifs 4 and 5). Motif 4 was characterized by a sustained high a1 amplitude, indicating that it is associated with turning behavior. Motif 5 showed a gradual increase in a1 amplitude, suggesting that it reflects the transition into a curved body posture (Fig. S5). Motif 5 showed a gradual increase in a1 amplitude, suggesting that it reflects the transition into a curved body posture (Fig. S5). We did not capture backward behavior motifs, possibly because our strain showed less long and continuous backward crawling than the wild-type strain.

The motif occurrences represented the animal's time-varying posture dynamics without obvious backward information. Therefore, we assigned forward, backward and pause labels (1, −1, 0) to each time point based on the velocity of the midpoint of the centerline as an additional feature. The pause state was identified using a kernel density estimation (KDE)-based thresholding method (see Materials and Methods, Fig. S1). Together with the seven motif occurrences from TDE-RICA, the total eight-dimensional features were used to describe the worm's behavior at each time point for the following analysis. Next, we projected the eight-dimensional vectors onto a 2D map for visualization and behavior state classification using t-distributed stochastic neighbor embedding (t-SNE) (Fig. 2B) (Berman et al., 2014; Liu et al., 2018). We initially generated the t-SNE map using the combined young and aged datasets; however, the young and aged groups occupied almost completely distinct regions in the resulting map, making direct comparison difficult (Fig. S6A). Moreover, compared with the t-SNE map generated using only the young dataset, the map generated from the combined young and aged datasets showed a lower density overlap between groups, as indicated by higher Jensen-Shannon divergence and Earth mover's distance. The combined dataset map also showed poorer cluster segmentation performance, as indicated by a lower silhouette score (Fig. S6B). Therefore, in this study, we generated the t-SNE map using the young dataset and then projected the aged animals into this behavioral space. The results revealed distinct locomotion patterns between young and aged groups. Watershed segmentation of the t-SNE map identified 11 clusters. Note that time frames that had been excluded from the TDE-RICA and t-SNE analyses for technical reasons were included in Fig. 2C as Clusters 0 and 12: frames exhibiting prolonged complex postures (which could be regarded as a turn behavior) were grouped into Cluster 0 (see Materials and Methods), whereas worms that ceased movement during the recording were assigned to Cluster 12. We assigned behavioral characteristics to each cluster by visually comparing the cluster IDs with the motif occurrences, locomotion velocity and original movies (Fig. 2C; Figs S7 and S8, Movies 3-6). Clusters 4, 7, 10, and 11 (red-toned colors in Fig. 2C and Fig. S8) were assigned as forward behavior states; Clusters 2 and 8 (blue-toned colors) were assigned as backward behavior states; Cluster 9 (yellow-toned color) was identified as slow forward movement. This cluster exhibited a lower forward locomotion speed than forward-related Clusters 4, 7, 10, and 11 (Fig. S8) and was not included in the forward locomotion loop (Movie 6), Fig. 2G. Clusters 5 and 6 (green-toned colors) were assigned as turn behavior states (Fig. 2D). We further observed a behavioral subtype within these clusters, which we identified as U-shape behavior (see Materials and Methods, Fig. 2E). Clusters 1 and 3 (pink-toned colors) were assigned as bending behavior states. Worms in these clusters often move forward with mildly complex body bending, in which the head or tail approaches or contacts the body, which often occurs before or after a turn (Fig. 2F).

To demonstrate that the identified behavioral clusters are not simply artifacts of the two-dimensional visualization, we performed an additional clustering analysis in the original high-dimensional behavioral feature space using k-nearest-neighbor (KNN)-Louvain clustering (see Materials and Methods). The resulting KNN-Louvain cluster labels were then projected onto the same t-SNE map only for visualization. We found that the KNN-Louvain clustering recovered the major behavioral regions identified by the t-SNE-watershed approach (Fig. S9A). KNN-Louvain labels form coherent regions on the t-SNE map rather than appearing randomly scattered, indicating that the structure identified in the original high-dimensional space is consistent with our method by t-SNE. This supports the interpretation that the major behavioral regions observed in the t-SNE map are not merely visualization artifacts. It is also clear to distinguish the forward, backward and turn clusters. Cluster 11 (light cyan in Fig. S9A) appeared in two distinct regions of this space, with one region located near Cluster 10 (cyan) and the other near Cluster 4 (red). Both regions, however, belonged to the forward-locomotion cycle. The exact cluster boundaries were not completely identical between the two methods, which were expected given their methodological differences. For example, Cluster 5 was separated into several distinct regions. We further quantified the agreement between the t-SNE-watershed-defined clusters and the KNN-Louvain clusters using the adjusted Rand index (ARI) (Hubert and Arabie, 1985) and normalized mutual information (NMI) (Vinh et al., 2010) (Fig. S9B). These results support the findings that the main behavioral clusters are robust and are not solely artifacts of the t-SNE projection.

Next, we examined if the forward-locomotion deficits observed in the aged worms could be captured by this method. We plotted the forward-movement cycles onto the t-SNE map and found that young worms exhibited more pronounced and repetitive cyclical patterns, forming tighter loops and clear periodicity. In contrast, aged worms showed fewer distinct cycles and more irregular, seemingly chaotic trajectories, suggesting that aging may reduce the stability and periodicity of their locomotion behavior (Fig. 2G; Movies 7 and 8, Fig. S10). In the t-SNE space, we observed two distinct patterns of irregular forward-locomotion cycles. One trajectory pattern followed a zigzag line, and the oscillation amplitude varied substantially within an apparent single cycle (i.e. across adjacent time points along the same loop), indicating nonuniform sinusoidal bending and reduced anterior-posterior coordination, consistent with uneven propagation of the body wave (Fig. 2H, lower; Movie 10). Another trajectory pattern intermittently exited the forward-locomotion clusters and transiently visited other clusters in the plots, although the corresponding movies suggested sustained forward locomotion (Fig. 2I, lower; Movie 12). We interpreted this as a subtle, fine-scale defect in motor control, manifesting as hesitations or micro instabilities during forward movement rather than a gross misclassification of the behavioral state (Fig. S11).

### Locomotor state transitions with Markov chain

In t-SNE space, forward-related clusters (4, 7, 10 and 11) exhibited periodic trajectories, which form a smooth forward movement. However, the trajectory patterns in the backward-related clusters (2 and 8) or the turning-related clusters (5 and 6) did not reveal any clear behavioral differences (Movies 4 and 5). Therefore, rather than focusing on the fine differences between clusters, we shifted to identifying broader behavior states, such as forward, backward and turn. Interestingly, we noticed that a subset of frames within ‘turn’ clusters represented atypical postures (Fig. 2E) that lacked a large change in movement direction typically found in a complete turn. And such discrepancy seems to be more prominent in the aged worms. To better analyze the discrepancy between young and aged worms, we incorporated body-axis angular velocity to subclassify turn events. Specifically, events within ‘turn’ clusters were identified as U-shape events when the angular velocity across the entire behavioral sequence did not exceed a predefined threshold (see Materials and Methods). This threshold allowed us to distinguish U-shape postures from complete turn events, while also excluding turn events that were interrupted by backward movement and failed to complete an actual directional change. As noted above, aged animals were more likely to cease movement during the latter half of the recording. Because the specific state from which an animal entered stopping was not the focus of this study, we excluded the stop state from all subsequent state-transition analyses. To emphasize switching dynamics rather than dwelling time, we ignored self-transitions by compressing consecutive identical states. The final states we used for the transition analysis are forward (F), backward (B), bending (E), slow forward (SF), turn (T) and U-shape (U).

**Fig. 3.**
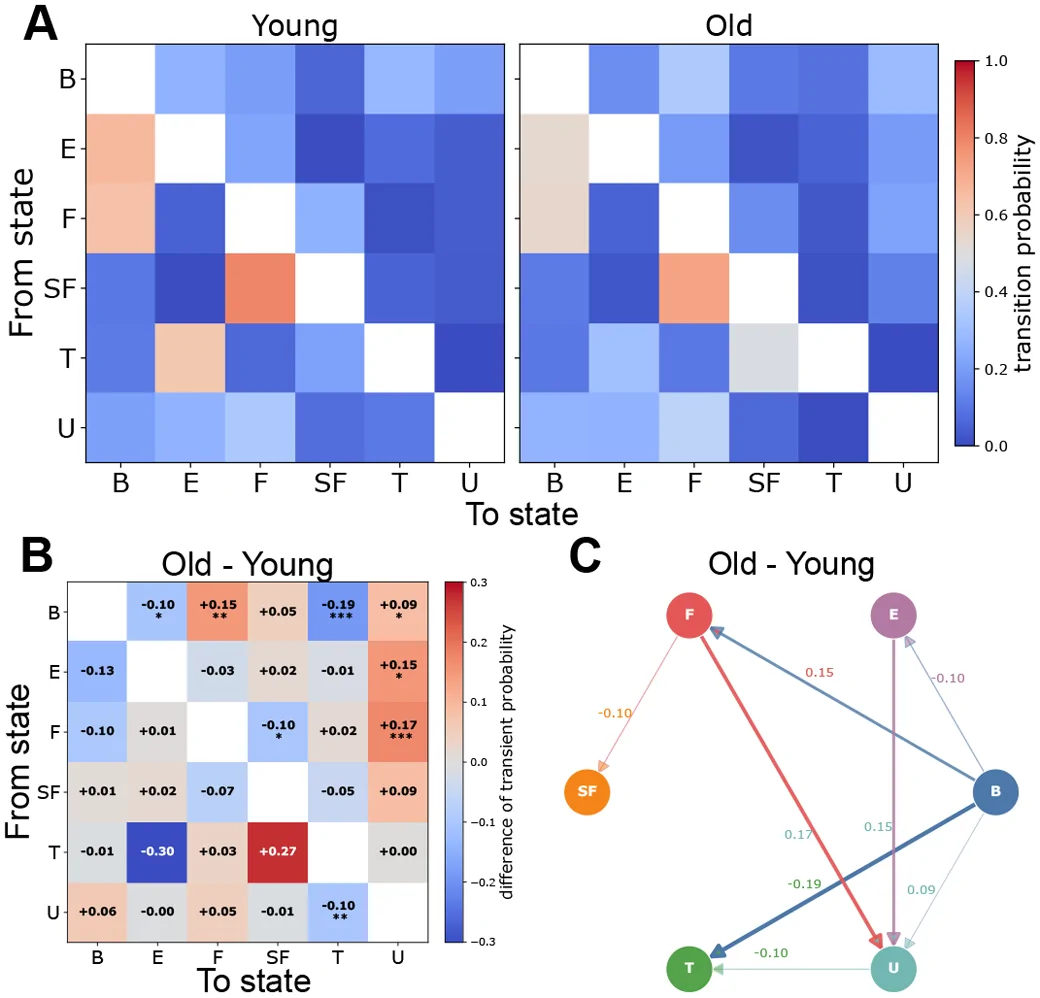
**Age-related changes in behavioral state transitions.** (A) Heatmaps show the transition probability matrices for the young and aged groups, and (B) the differences between the two groups are plotted as Old - Young. Rows indicate the from-state and columns indicate the to-state. Mann-Whitney *U* test, *P<0.05, **P<0.01, ***P<0.001. The statistical analysis was performed at the level of individual worms. Sample sizes were *n*=16 worms for the young group and *n*=16 worms for the aged group. (C) Directed network representations of the Old - Young matrix, where nodes denote states and edges represent transitions that differed significantly between the young and aged animals. Edge thickness and transparency reflect transition strength.

Group-specific transitions were visualized as heatmaps (Fig. 3A), and the difference matrix (Old - Young) was plotted to highlight group differences (Fig. 3B). To facilitate interpretation at the network level, we also represented the transition matrix as directed graphs in which nodes correspond to behavior states and edges represent transitions (Fig. 3C).

The difference map revealed that backward to U-shape (B-U) or forward (B-F), bending to U-shape (E-U) and forward to U-shape (F-U) were strengthened in aged animals relative to the young, while backward to bending (B-E) or turn (B-T), U-shape to turn (U-T) and forward to slow forward (F-SF) were weakened. The Old - Young network representation highlighted transitions with significant group differences, which were concentrated on a limited number of edges within the transition network (Fig. 3C). These results provide a summary of how aging reshapes behavior-switching patterns.

Specifically, we identified that the transition from or to backward-movement states showed significant differences between the two groups. Backward followed by a turn (B-T), which resulted in a larger change in movement direction, is common and produces effective exploration or escape behaviors in worms (Gray et al., 2005; Hums et al., 2016). We, therefore, next examined whether this B-T behavior differs systematically between groups in the following part.

### Decreased turn behavior following backwards in the aged animals

**Fig. 4.**
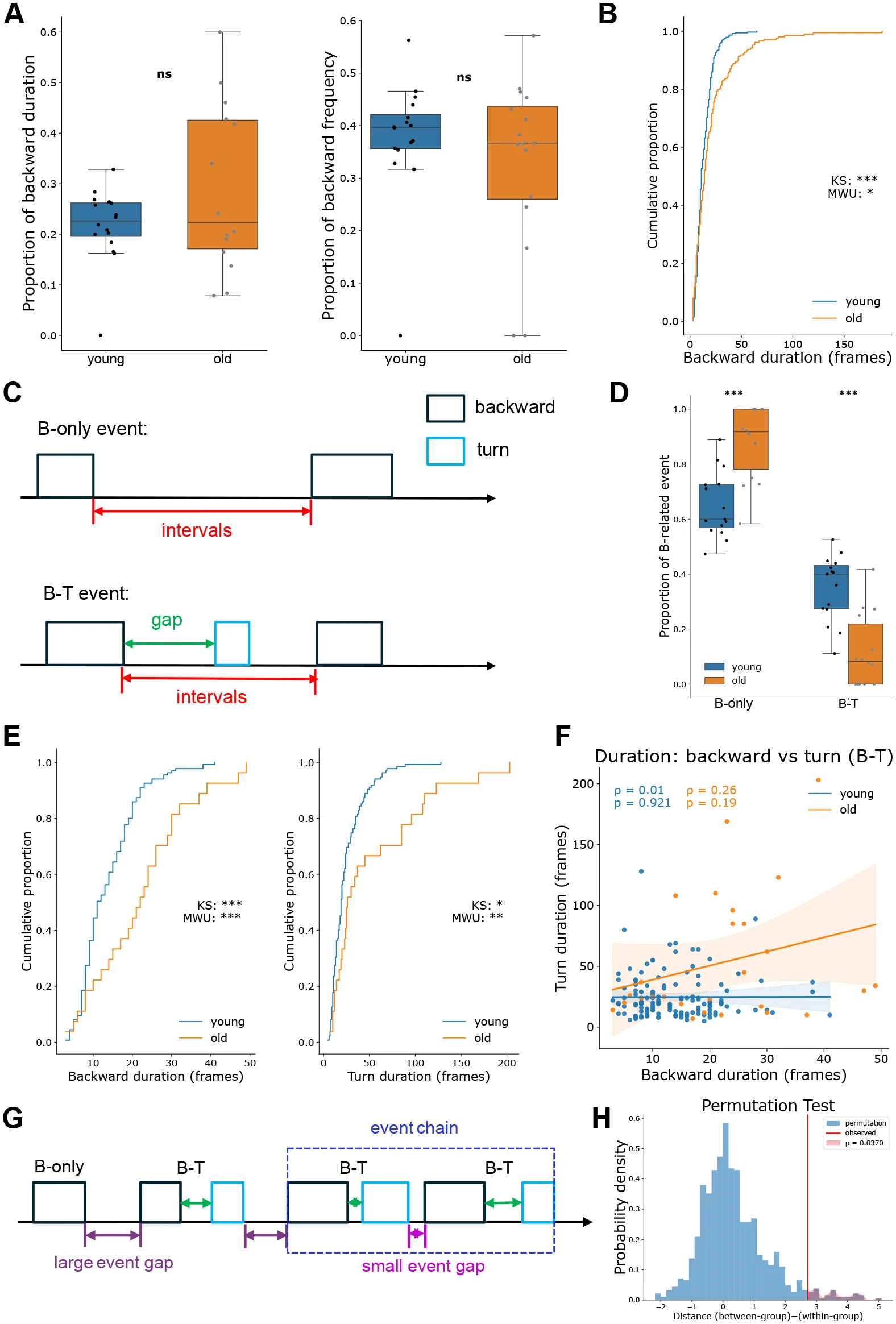
**Age-related changes in backward- and turn-related behaviors.** (A) Proportions of backward-related events. Fraction of total recording time spent in backward locomotion (left) and the frequency of backward events (right). (B) Empirical cumulative distribution functions (ECDF) of backward duration. The curves show the cumulative probability of backward durations for young and aged worms. (C) Schematic definition of backward event patterns. Frame-wise cluster labels were used to segment backward and turn behaviors into two distinct patterns. B-only: A backward event that is not followed by a turn. B-T: A backward followed by at least one turn. (D) The fraction of B-only and B-T events among all backward events. (E) ECDF of backward duration (left) and turn duration (right) for the B-T events. (F) Relationship between backward and turn duration in B-T events. Each point represents one event pair, and *p* represents Spearman's rank correlation. (G) Schematic definition of chaining the backward- and turn-related events. (H) Permutation test of DTW-based event chain shape separation between groups. For panels A and D, significance was determined using a Mann-Whitney *U* test; ***P<0.001. Boxes indicate the interquartile range, centerlines indicate the median, and whiskers extend to 1.5× the interquartile range. And for panels B and E, distribution shapes and central tendencies were evaluated using two-sided Kolmogorov-Smirnov and two-sided Mann-Whitney *U* tests, respectively; *P<0.05, **P<0.01, ***P<0.001. For panel H, group separation of event chain shapes was assessed via a permutation test (1000 iterations); the observed value (red line) represents the difference between the mean between-group and within-group DTW distances against the permuted null distribution (histogram), *P*<0.05. Sample sizes were *n*=16 worms for the young group and *n*=16 worms for the aged group.

We first examined whether the overall time duration and frequency of backward behavior exhibited any difference in the two groups. However, we did not observe a significant difference in either metric (Fig. 4A), though the aged worms had a longer single backward duration (Fig. 4B). This indicated that aged animals may occasionally show long but rare backward movements, which were not revealed by averaging.

We then classified backward patterns into two categories, backward only (B-only) and the B-T behaviors, based on whether a turn event occurred between two consecutive backward events (Fig. 4C, see Materials and Methods). Compared with young worms, aged animals exhibited a higher proportion of B-only events (Fig. 4D; Table S1). This observation is consistent with the state-transition matrix analysis, which showed that turn events were less likely to occur following backward behaviors in aged worms (Fig. 3A,B). We next investigated the time duration of backward and turn behaviors in the B-T event and found that the aged animals tended to exhibit longer backward and turn movements (Fig. 4E). There is no obvious relationship between the backward duration and the turn duration in young or aged worms (Fig. 4F).

We noticed that some of the B-T events occurred in close succession, with little or no temporal separation, and during such sequences, animals appeared to show a variation of postural changes. We therefore next constructed event chains by merging temporally proximal events into a single episode (Fig. 4G). We defined merged chains as those containing at least one turn behavior. We then analyzed the structure of each chain by using motif occurrences and compared the temporal shape of these chains between the two groups using the dynamic time warping (DTW)-based permutation test. DTW analysis revealed that the chain trajectories are more similar within groups than between groups, as indicated by an extremely large observed distance in Fig. 4H, which also exceeded the permutation null values. This result suggested a group-specific dynamics in the backward-turn-related event chains.

### Increased individual differences with aging

The above results demonstrated that the aged group exhibited overall locomotion deficits. Nevertheless, some aged individuals, as well as certain time points within their trajectories, still displayed movement patterns that more closely resembled those of the young group. This observation highlights the presence of individual variability and raises the possibility of ‘healthy aging’ within the aged population (Cho and Park, 2024; Cho et al., 2025; Freund, 2019). To quantitatively describe these individual differences, we performed a detailed analysis of the behavior cluster occupancy and the Shannon entropy of behavior state distributions (see Materials and Methods). These metrics allowed us to assess the degree to which each individual deviated from its group average and to determine whether the heterogeneity within the aged group was significantly greater than that of the young group.

**Fig. 5.**
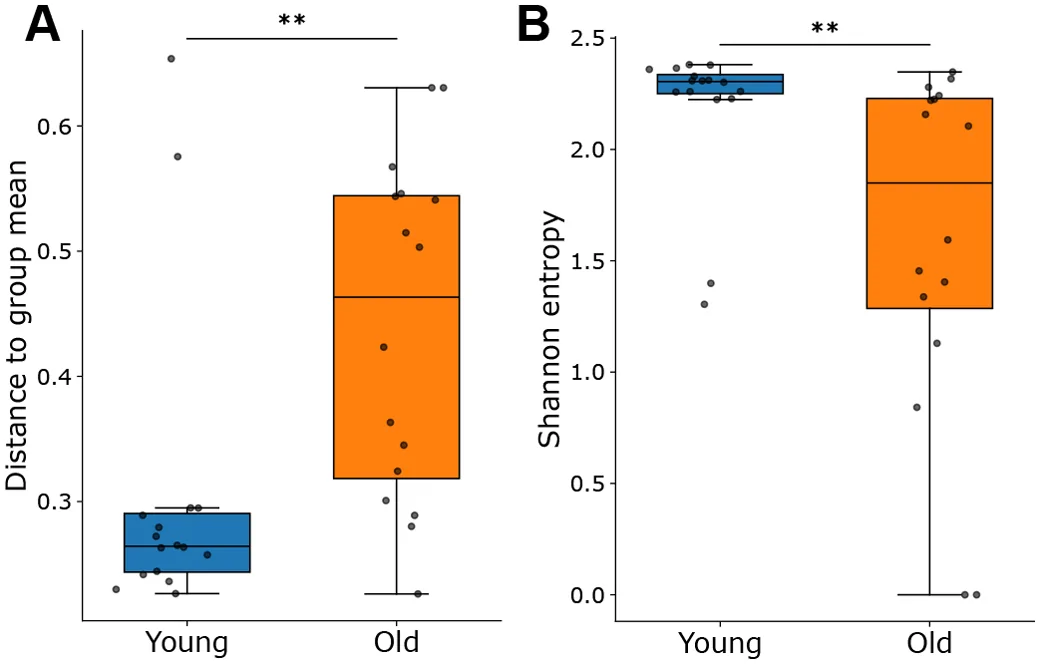
**Individual variability in behavioral pattern usage.** (A) Dispersion of behavior cluster occupancy across groups. The JSD indicates the distance between each individual's behavior cluster occupancy and the group mean. (B) Shannon entropy of behavior cluster distributions. A higher entropy reflects more uniform usage of behavioral states. Each dot represents an individual, Mann-Whitney *U* test, **P<0.01. Boxes indicate the interquartile range, center lines indicate the median, and whiskers extend to 1.5× the interquartile range. The statistical analysis was performed at the level of individual worms. Sample sizes were *n*=16 worms for the young group and *n*=16 worms for the aged group.

As shown in Fig. 5A, the aged group showed significantly greater dispersion in behavior cluster occupancy compared with the young group, as quantified by the Jensen-Shannon distance (JSD) to each group's mean profile. The JSD provides a symmetric and bounded measure of dissimilarity between probability distributions, with higher values indicating greater deviation from the group-level behavior patterns. Shannon entropy values revealed that aged individuals showed lower and more distributed entropy values, indicating that their behavioral repertoire was more restricted to variable extents among individuals (Fig. 5B). Together, these findings quantitatively captured increased variability in aged animals.

## DISCUSSION

In this study, we used an embedding-based behavior time-series framework to characterize behavior decline in *C. elegans* at a fine-grained level. Our results show that aging-associated locomotion defects cannot be fully captured by rough measures alone, such as the total duration or frequency of behavior states. Instead, the major aging-related declines became evident at the level of behavior-transition architectures. The transition from backward to turn behavior emerged as one of the most prominent features to distinguish between young and aged animals. Turn movement, which often occurs following backward locomotion, is generally regarded as reorientation behavior and plays an important role in avoidance responses. In this context, the increased frequency of B-only events and the reduced proportion of B-T in the aged animals indicate a weakening ability in reorientation. Impaired reorientation could affect behaviors such as chemotaxis (Iino and Yoshida, 2009), thermotaxis (Mohammadi et al., 2013) and navigation (Gray et al., 2005), potentially reducing the worms' ability to respond appropriately to environmental cues. Importantly, this finding does not appear to be strain specific, as similar results were also observed in wild-type animals (Figs S12 and S13).

Episodes consist of consecutive short runs, and associated turns form the basis of pirouettes, which is an effective strategy used when worms move down an attractant gradient (Pierce-Shimomura et al., 1999). We also observed that backward- and turn-related events often occurred in close temporal succession and could be merged into extended behavior chains. This forms a higher level of behavior organization. Group differences in these chains indicate that aging affects not only isolated transition states but also the broader sequential events. This phenomenon was observed in an off-food environment without external stimuli, suggesting the possibility that complex behaviors resembling pirouettes may occur even under naturalistic conditions.

Forward locomotion also exhibited distinct aging-related abnormalities. We identified two specific irregular forward-locomotion phenotypes. First, there is nonuniform trajectory across consecutive time points. Second, forward locomotion transitioned more frequently into other behavior clusters. These results indicate that, in the aged animals, the dorsal and ventral sinusoidal waves become less coordinated and less periodic. Consistent aging-related defects in forward locomotion were also observed in wild-type animals (Figs S14 and S15). Such rhythmic motor behaviors are generated centrally [central pattern generators (CPGs)], which do not require sensory feedback or other patterned inputs (Katz, 2016; Marder and Bucher, 2001). Moreover, the general neural organization of CPGs subserving locomotion appears to be quite similar in invertebrates and vertebrates (MacKay-Lyons, 2002). Therefore, this instability observed in the aged worms may resemble the increased gait variability observed in aged humans, such as irregular stride length and rhythm, and suggests that aging is associated with impaired stability of motor control. More importantly, these findings indicate that aging does not simply reduce locomotion speed but also alters the temporal coordination among behavior modules. Previous studies have demonstrated that highly consistent behavioral sequence patterns were observed across species (Minasandra et al., 2025). Therefore, this study may also provide potential insights into behavioral aging in mammals, including humans.

Traditional analytical measures, such as locomotion speed or specific state frequency, may overlook changes in behavioral sequential structure and transitions. The analytical framework used in this study captures aging defects at the levels of subsequence structure, revealing defects that remained hidden in previous analyses (Glenn et al., 2004) and suggesting that aging-related behavior changes may be better understood as a functional decline in how motor systems are temporally linked and organized.

The behavior defects identified in this study may also provide useful insights for linking aging-related locomotion declines to brain-wide neural dynamics. The destabilization of forward-locomotor periodicity and the weakened coupling between backward and turn movements may reflect age-dependent functional degenerations in neural dynamics. Previous studies have suggested that the dynamics of many interneurons and motor neurons represent underlying motor commands even in physically or chemically immobilized worms lacking motor feedback (Kaplan et al., 2018) and that these motor commands can be described within a low-dimensional manifold represented by distributed patterns of neural activity (Kato et al., 2015). In parallel, locomotion in *C. elegans* has been proposed to follow a three-timescale behavioral hierarchy implemented by nested neuronal dynamics, in which slow brain-wide activity gates faster motor neuron oscillations and switches them between distinct functional modes (Kaplan et al., 2020). Future studies should combine behavior analysis with whole-brain imaging in aged animals, which would be essential for establishing how aging reshapes the relationship between distributed neural dynamics and behavioral organizations.

This study presents a quantitative analysis of age-associated behavioral defects in *C. elegans* and addresses an important question in aging biology. We hope that this work could serve as a bridge linking the fields of aging and neuroscience. However, there are several limitations of this study. First, although Markov chain analysis provides a useful framework for quantifying the state transitions, it has intrinsic limitations (Sanabria et al., 2019). In particular, this model assumes that the next behavioral state mainly depends on the current state, while real animal behavior may be influenced by longer behavioral history and internal physiological states. In addition, discretizing continuous behavior into a limited number of states may simplify complex behavior dynamics. Second, although we reported that there are some intervals in B-T behavior, the functional significance of the interval remains unclear. Further experiments are required to clarify whether this interval may reflect a decision-making or information integration period. In addition to the DTW-based analysis, other features of the backward- and turn-related event chains may provide further insight into how aging affects reorientation behavior. Third, this study mainly focused on TOY21 and wild-type strains. Although the main conclusions were consistent between the two strains, some discrepancies remained, especially in backward duration (Fig. 4A,B,E; Fig. S13A,B,D). These discrepancies may reflect strain-specific differences, as the TOY21 strain carries multiple fluorescent markers for whole-brain imaging and may therefore differ from wild-type animals in locomotor behavior. Future analyses using additional genetic backgrounds, including mutants, will be important. Moreover, our current analysis focused on global locomotor states, including forward, backward, and turns. However, turn behavior itself includes distinct subtypes, such as delta turns and omega turns, and whether these subtypes are differentially affected during aging remains unknown. Finally, the TOY21 strain was generated to enable whole-brain imaging and neuronal annotation; future studies should integrate age-associated behavioral defects with neural activity patterns. Linking behaviors to whole-brain neural dynamics will be essential to understand how the nervous system loses the ability to generate appropriate behavioral patterns with aging.

## MATERIALS AND METHODS

### Cultivation of *C. elegans*

*C. elegans* was cultivated at 20°C on standard nematode growth medium (NGM) plates using standard methods (Brenner, 1974), seeded with *Escherichia coli* OP50 as food. *E. coli* OP50 was cultivated on Luria-Bertani (LB) agar plates and prepared in LB broth at 37°C overnight and then spread on NGM plates. Several worms were transferred to a new NGM plate every 4 days to avoid starvation and incubated until the next generation grew up. All strains used in this study are shown in Table 1.

**Table 1.** Strain list

Strain Name	Genotype	Source	Used in
N2	Wild type	CGC	Figs S12-S15
TOY21	*qjIs11[glr-1p::svnls2::TagBFPsyn,ser-2(prom2)p::svnls2::TagBFPsyn]; peIs3042[eat-4p::svnls2::TagRFP675syn,lin-44p::GFP]; Is[H20p::nls3::tdTomato,H20p::nls2::GCaMP6f]#72-2-5*	This study	All

### Selection of young and old *C. elegans*

One milliliter of bleaching buffer [prepared by mixing 1 N NaOH and household bleach (Kao Bleach Haiter) at a 5:2 volume ratio] was added onto NGM plates when there were large amounts of eggs in adult *C. elegans*. After a large number of eggs were released, the suspension was centrifuged, and the supernatant was removed. The remaining 10 μl of egg-containing suspension was transferred to a new NGM plate. Worms are sensitive to the bleach buffer and will be ruptured while the embryos are protected by their eggshells (Porta-de-la-Riva et al., 2012). This method provides a synchronized culture of worms. 3 days later, about 20-30 L4 worms (with a clear white half circle in the center of the ventral side, known as developing vulva) were picked on a new NGM plate. After 24 h, these worms were regarded as ‘1-day adults’ and were used for imaging in this research as the young group. Nine days after picking L4 larvae, the worms were regarded as ‘9-day adults’ and were used for imaging as the aged group. The worms were transferred to a new plate every 2 days to avoid confusion between newly born larvae and the selected worms during 9-day adults' cultivation. In this study, all analyses were performed using 16 young and 16 old worms collected from multiple batches.

### Behavior assay

To quantify the behavior of *C. elegans*, we performed a behavioral assay for both the young and aged groups. Adults were washed from growth NGM plates in the M9 buffer and transferred to a food-free NGM plate to allow worms to crawl freely. To minimize aggregation of worms and improve tracking reliability, approximately ten worms were transferred to each assay plate in each trial. After a 1-min recovery from the stimulus of transfer to the assay plate, worms were imaged for 5 min at 5 fps. The tracking system consists of a camera (Lw575M-IO; ARGO) and a LED illumination (HPR-150SW; CCS) equipped with a digital power source (PD2-3024; CCS) (Yoshida et al., 2012).

### Statistical analysis

#### Extract centerlines

Behavior images containing multiple worms were first cropped into individual-worm images using a custom MATLAB script (Mori et al., 2022). The binarization threshold of the images was determined manually using ImageJ, and the binarized images were submitted to WormTracer (Kuze et al., 2025) to extract worm centerlines. Centerlines in frames with complex worm shapes, such as coiling, were manually corrected to ensure accuracy. Frames that were too ambiguous to be resolved manually were labeled as Not-a-Number (NaN), and ignored in the following analysis.

#### Forward-locomotion phase, frequency and amplitude

Eigenworms were derived as previously described (Ahamed et al., 2021; Broekmans et al., 2016; Stephens et al., 2008). The forward-locomotion phase was computed from the second- and third-eigenworm coefficients a2(t) and a3(t) as *θ*(*t*)=*unwrap*(*arctan*2(*a*3(*t*), *a*2(*t*))). Instantaneous angular frequency was estimated by *ω*(*t*)=*dθ*/*dt* and smoothed using a Savitzky-Golay filter. Instantaneous frequency was *f*(*t*)=*ω*(*t*)/(2*π*) and summarized by its median or mean values; corresponding periods were computed as *T*=1/*f*. Oscillation amplitude was quantified as 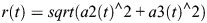. As complementary estimates of rhythmic timescales, we extracted the dominant period from the first non-zero-lag peak of the normalized autocorrelation function (*lags*>*τmin*) and the dominant frequency from the peak of the Welch power spectrum.

#### Phase coupling

Phase coupling between eigenworms a2(t) and a3(t) was quantified by the Hilbert-based instantaneous phase. Analytic signals were obtained via the Hilbert transform, and instantaneous phases were defined as *φa*(*t*)=*arg*(*H*{*a*(*t*)}). The phase difference was computed as Δ*φ*(*t*)=*arg*(*exp*(*i*(*φ*_*a*2_(*t*)−*φ*_*a*3_(*t*)))). The phase locking strength was summarized by the mean resultant length *R*=|〈*exp*(*i*Δ*φ*(*t*))〉|, and the mean phase offset was *μ*=*arg*(〈*exp*(*i*Δ*φ*(*t*))〉).

#### Pause state

The pause state was defined based on the distribution of locomotion velocity (Fig. S1A,B). A one-dimensional Gaussian KDE was applied to estimate the underlying probability density of the velocity distribution (Fig. S1C,D). Multiple local maxima in the KDE curve were detected using a peak-finding algorithm. Based on these KDE-derived peaks, two local maxima surrounding the low-velocity region were selected as the lower and upper bounds of the pause-state interval. Frames with velocity values falling within this interval (red dashed lines in Fig. S1C,D) were classified as pause-state frames.

#### TDE-RICA

As the young and aged worms have different locomotion undulation frequency, to ensure the two groups share the same undulation frequency in forward locomotion, the young data were resampled by interpolation before time-delay embedding. Specifically, young worm data were interpolated along the time axis by using piecewise cubic Hermite interpolating polynomial interpolation, thereby reducing locomotion frequency to approximately match that of aged worms. We compared the original and resampled young datasets by performing TDE-RICA and projecting the results onto t-SNE maps. The extracted motifs and state classifications were largely consistent between the two datasets, indicating that interpolation did not substantially affect motif extraction or behavioral state classification (Fig. S4).

The resampled young data were then pooled with the aged data and jointly subjected to TDE-RICA. Given a recording time series of T frames, the matrix should be *M*=5*T, *M*∈*R*^5×*T*^ (occurrences of five eigenworms), the embedded subsequence at time point *t* is 5*M(*t*: *t*−1+*K*), *t*∈(1:*t*−*K*+1), and the embedded time series of sample *i* of matrix *M* is *Xi*=(5*K*)* (*T*−*K*+1), where *K*=25 (Ahamed et al., 2021).

Reconstruction ICA was performed to capture elementary behavior motifs as a previous report (Toyoshima et al., 2024). Independent components are *M*=5K*m, where *m* is the number of motifs. Here, we chose *m*=7 as previously described (Ahamed et al., 2021). The amplitude matrix of sample *i* is as follows:

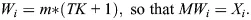


#### Behavioral clusters defined by t-SNE-watershed clustering

To generate a behavior state map, we added an additional feature that labels the forward, backward and pause as 1, −1, and 0, respectively. This was obtained based on the velocity of the midpoint of the backbone. The total eight features (seven TDE-RICA amplitudes plus the forward/backward/pause label) from the young animals were then reduced to a two-dimensional representation using the nonlinear dimensionality-reduction method t-SNE (Berman et al., 2014; Cieslak et al., 2020; Liu et al., 2018; Mori et al., 2022). 11 clusters were classified by the watershed method, and each cluster was assigned to a behavior state manually. At each time point, the worm belonged to a specific behavior cluster. Data from aged animals were subsequently projected onto the t-SNE map generated from the young dataset. For further analysis, points with a residence time of fewer than three frames in a given cluster were reassigned to the preceding cluster label.

#### KNN-Louvain clustering

To validate the t-SNE-watershed-defined behavioral clusters, we performed an additional clustering analysis using the original high-dimensional behavioral features. The feature matrix was first standardized, and a KNN graph was constructed based on distances between time points in the original feature space. The graph was symmetrized and used as the input for Louvain community detection. The resulting KNN-Louvain cluster labels were then projected onto the same t-SNE map for visualization and comparison. To quantify the agreement between the t-SNE-watershed-defined clusters and the KNN-Louvain clusters, we calculated the ARI and NMI.

#### Behavioral state transitions based on Markov chain

For each animal, we compressed the frame-wise state sequence into a single occurrence, thereby emphasizing transition structure and disregarding self-transitions during dwelling in the same state. Transition counts were computed by tallying adjacent state pairs across individuals within a group rather than estimating per-animal matrices. We then accumulated a transition count matrix *C*, where *C_ij_* denotes the number of observed transitions from states *i* to *j*. To avoid zero-probability entries, we applied a small additive pseudocount 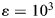. The transition probability matrix was calculated as 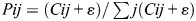.

#### Calculation of the body-axis angular velocity

We calculated angular velocity along the body axis to distinguish turn behaviors and long-lasting C shapes. For each frame *t*, we defined the body-axis vector as *u*(*t*)=(*ux*(*t*),*uy*(*t*))=(*x*0(*t*)−*x*1(*t*),*y*0(*t*)−*y*1(*t*)), where (*x*0,*y*0) and (*x*1,*y*1) denoted two endpoint coordinates of the centerline (head and tail), and its corresponding orientation angle was *θ*(*t*)[*rad*]=*atan*2(*uy*(*t*), *ux*(*t*)), *θ*∈(−*π*, *π*]. Frames for which the centerline assignment could not be resolved were excluded from the analysis. Finally, the angular velocity along the body axis was calculated as *ω*(*t*)[*rad*/*s*]=*unwrap*(*θ*(*t*+Δ*t*)−*θ*(*t*))/Δ*t*, where Δ*t* is the time interval between two consecutive valid frames. U-shape events were defined as events in which the angular velocity did not exceed a data-driven threshold, defined as the 95th percentile of the absolute angular velocity distribution.

#### Extraction of backward and related movement events

We extracted backward movements and their subsequent turns from frame-wise behavior state labels. The procedure consisted of three steps.

1. Backward segmentation

All frames labeled as backward were identified and grouped into contiguous backward segments. Each segment was represented by its start- and end-frame indices. These backward segments served as anchors to define the temporal windows for searching subsequent turn behavior.

2. Event classification between consecutive backward segments

For each backward segment *k_i_*, we examined the interval starting immediately after its end and the start of the next backward segment *k_i_*_+1_ (‘interval’ in Fig. 4C, or the end of the recording for the last backward segment). Within this interval, frames labeled as turns were grouped into contiguous turning segments. Based on the presence and features of turn segments, we recorded the following:

2.1. backward-only events when no turn frames occurred before the next backward event;
2.2. backward-turn events when at least one turn segment occurred after a backward bout. For each event, we summarized the turn block by the onset of the first turn frame and the end of the last turn frame and recorded the number of turn segments and the intervening gap between backward offset and turn onset (‘gap’ in Fig. 4C).

3. Merging repeated backward-turn sequences

To capture prolonged complex behavior patterns composed of repeated backward-turn events, we optionally merged temporally adjacent backward-turn events into chains when the gap (‘event gap’ in Fig. 4G) between consecutive events did not exceed 20 frames in the young group or 17 frames in the aged group. This was determined from the distribution of event gap durations. Chains were retained only if they contained at least one turn behavior, ensuring that the merged events specifically reflected backward-turn patterns rather than backward-only events. Each merged chain was recorded by its temporal span, the list of reversal bouts, the list of turn bouts, and the gaps between them.

#### DTW and permutation test

To quantify group differences in the shape of backward-turn-related chains, we computed the event chain using DTW. Each chain segment was represented as a matrix *X*∈T*7 (frame length *T* with seven-dimensional motifs). DTW alignment was constrained by a window *w* so that frame *i* in one segment could align only to frame *j* in the other segment when |*i*−*j*|≤*w*. The local matching cost was defined as the Euclidean distance. The final DTW distance was normalized by (*Tx*+*Ty*) to reduce scale dependence on segment length. We computed the difference between the inter-group and intra-group mean DTW distances as Δ*D*=D_*inter*_−D_*intra*_. Statistical significance was assessed using a permutation test that randomly permuted group labels at the worm level, and Δ was computed for each permutation; 1000 iterations (*n*) were performed. The *P*-value was computed as 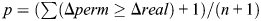 to test whether the observed between-group separation exceeded by chance.

#### Behavioral cluster occupancy and entropy analysis

Behavioral cluster labels were obtained from the t-SNE map described above. The occupancy of each cluster was then calculated as the fraction of frames assigned to that cluster, generating an occupancy probability distribution for each individual worm. To quantify individual variability in behavioral cluster usage, we first calculated the mean occupancy profile within each group and then computed the JSD between each individual occupancy profile and the corresponding group mean profile. To quantify the diversity of behavioral states used by each worm, Shannon entropy was calculated from the cluster occupancy distribution of each individual, which was defined as follows:

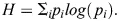
where *p*_*i*_ represents the occupancy probability of cluster *i*.

## Supplementary Material



10.1242/biolopen.062711_sup1 Supplementary information

Table S1. List of the numbers of backward- and turn-related behavioral events
