# Supplementary material for: Aging-related behavioral defects in Caenorhabditis elegans
Source: Biol Open. 2026 Aug 17;15(8):bio062711. doi: 10.1242/bio.062711 (PMC13552477; doi:10.1242/bio.062711)
Supplement: Supplementary file 1 [file biolopen-15-062711-s1.pdf]

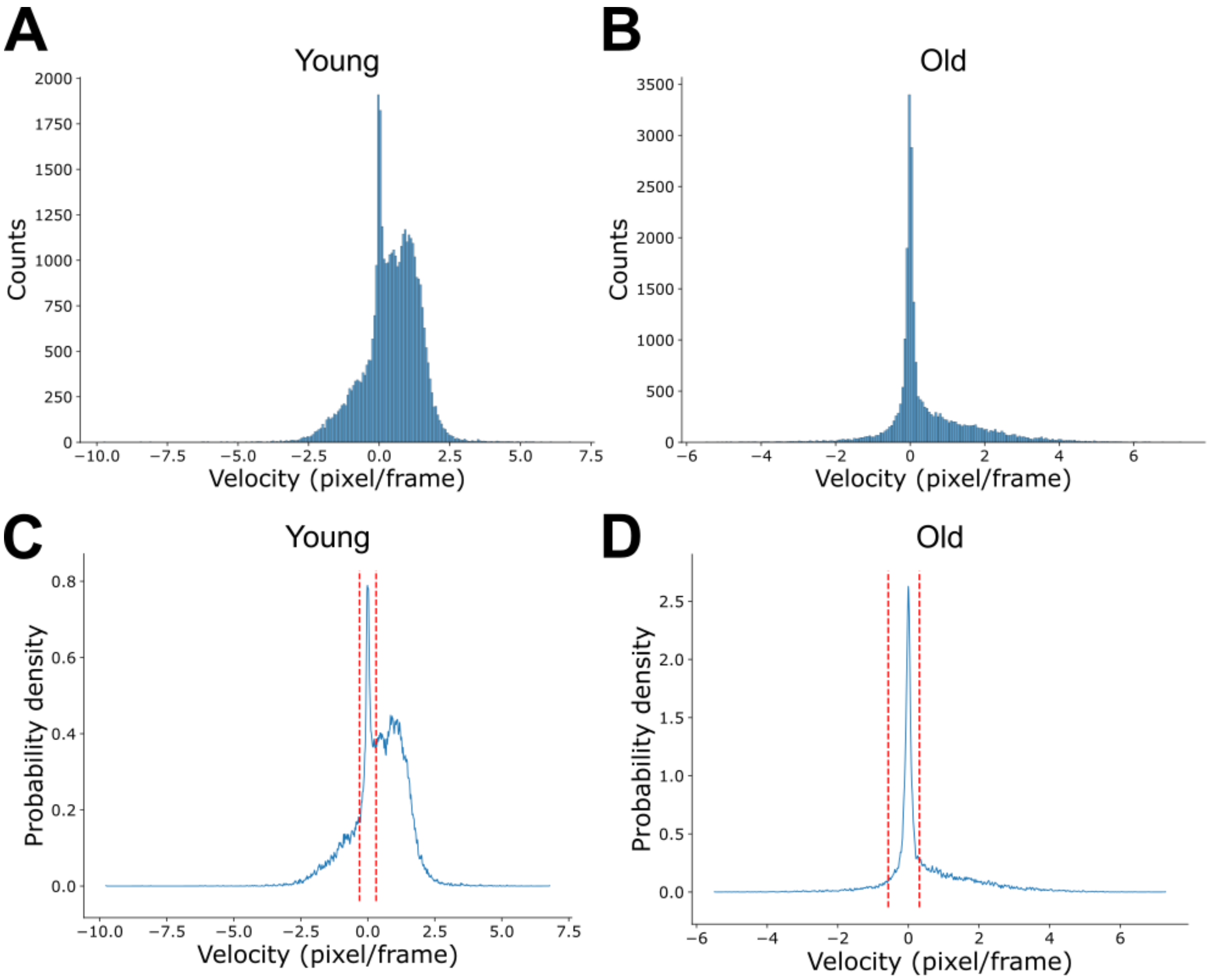


**Fig. S1. Classification of locomotion states based on velocity distribution.**

(A, B) Velocity histogram and (C, D) the corresponding 1D Gaussian KDE in young and aged datasets. Multiple local maxima were detected from the KDE curve. The two red dashed lines indicate the manually selected boundaries that divide the velocity distribution into reversal, pause and forward states. Sample sizes were n = 16 worms for the young group and n = 16 worms for the aged group.

Biology Open • Supplementary information

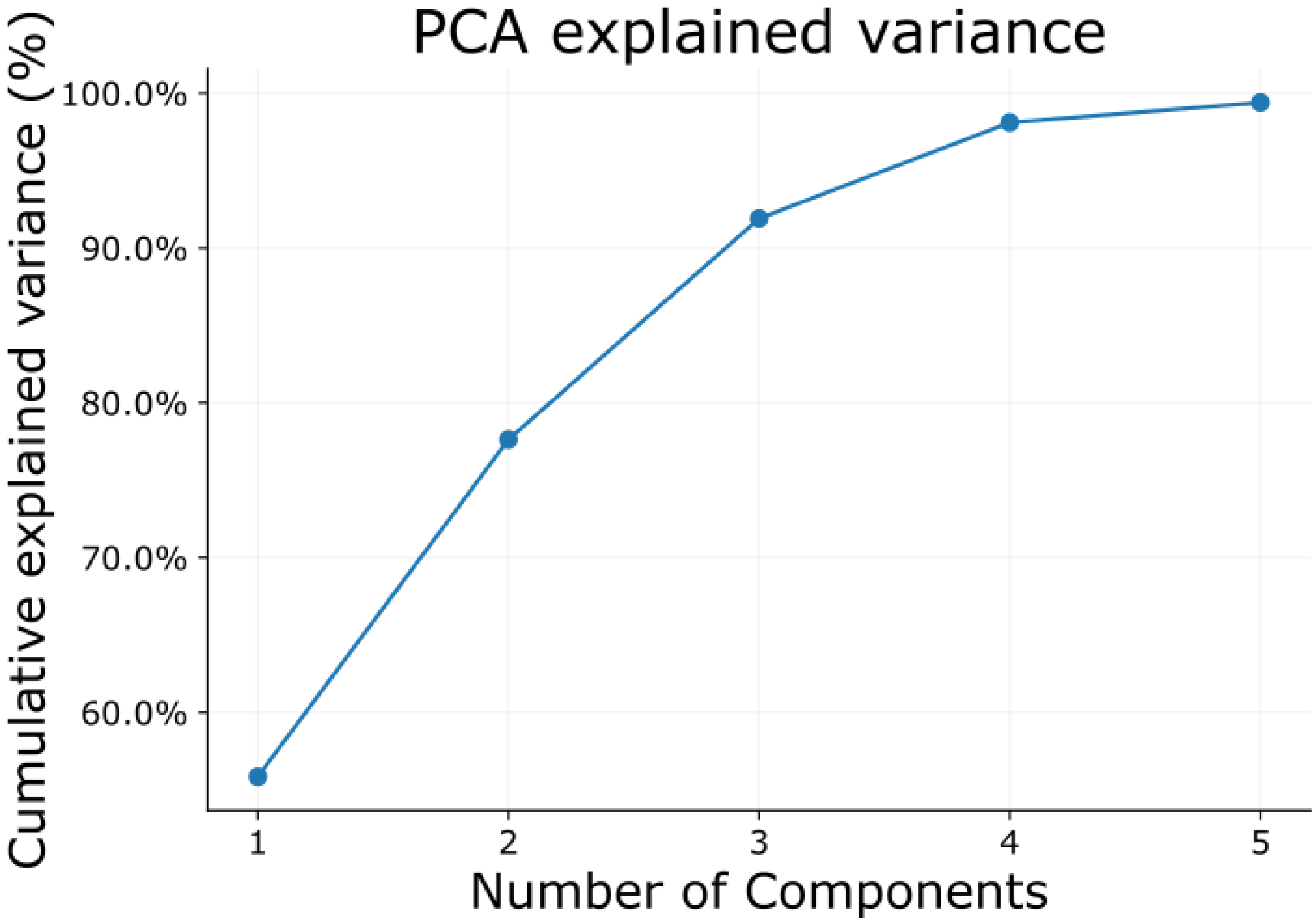


**Fig. S2. Cumulative explained variance of the PCA components.** The top five components captured more than 99% of the total variability.

Biology Open • Supplementary information

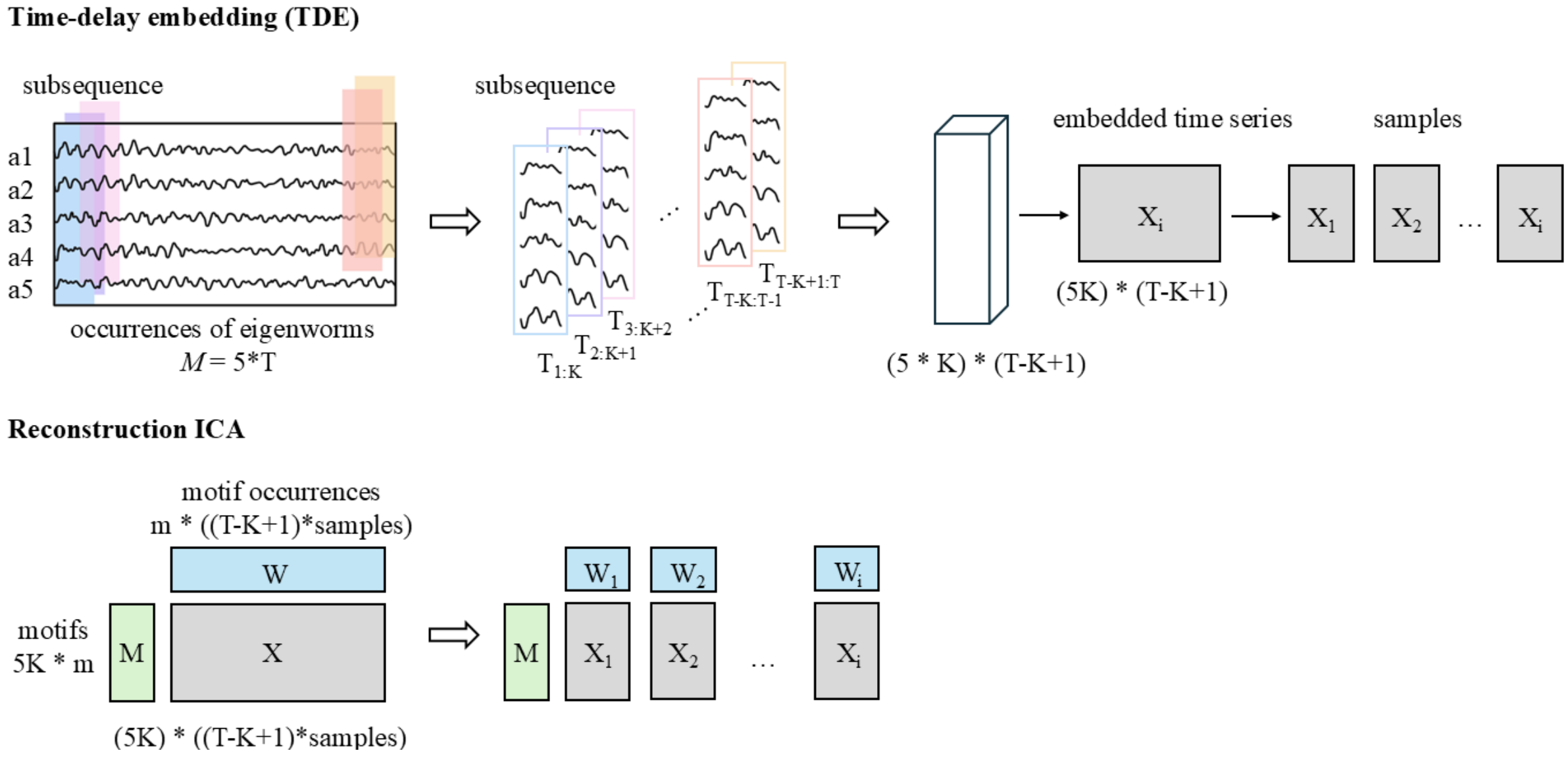


**Fig. S3. Graphical representation of TDE-RICA.**

For each time point, a subsequence of length $K$ was extracted from the 5-dimensional eigenworm occurrence series and concatenated to form an embedded vector with $5K * (T - K + 1)$ shapes. The embedded vectors from all samples were then assembled into the embedded time-series matrix $X$. Reconstruction ICA was applied to this matrix $X$ to decompose the embedded behavioral dynamics into a set of motifs $M$ and their corresponding temporal occurrences $W$. The motif matrix $M$ represents elementary behavioral patterns, whereas $W$ represents the occurrence strength of each motif across time.

Biology Open • Supplementary information

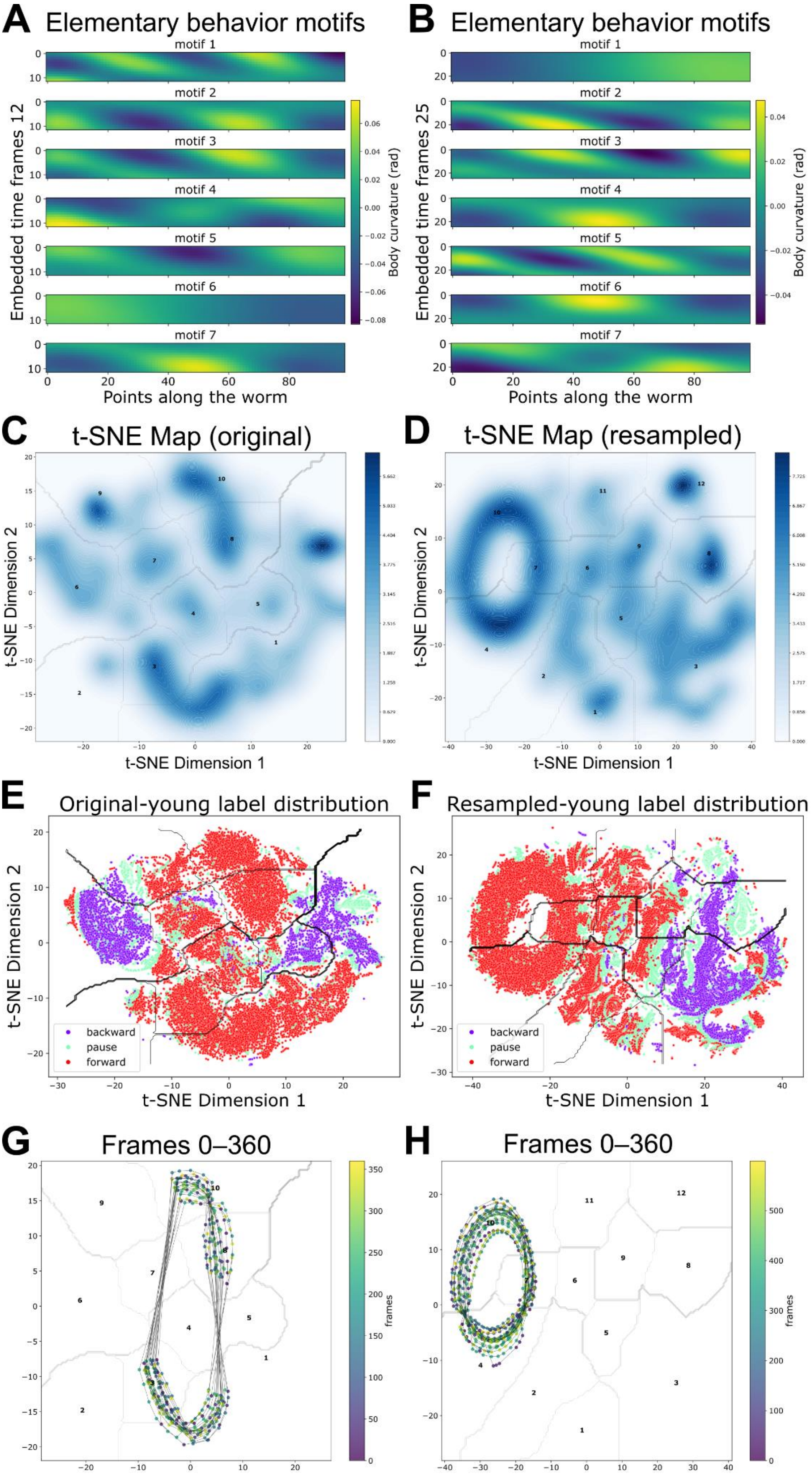


Biology Open • Supplementary information

**Fig. S4. Locomotion behavior classification based on t-SNE and watershed clustering using the original and resampled young datasets.**
For all pairs, panels on the left (A, C, E, G) represent the original young dataset, and panels on the right (B, D, F, H) represent the resampled young dataset. (A, B) Elementary behavioral motifs. The x-axis represents body position, and the y-axis represents embedded frames. (C, D) Probability density maps in t-SNE space (E, F) Distribution of forward, backward, and pause labels on the t-SNE maps, where each dot represents a single time point. (G, H) Representative forward movement trajectories on t-SNE map. Each colored trajectory corresponds to a continuous time-series movement, with specific time frames indicated in the panel title.

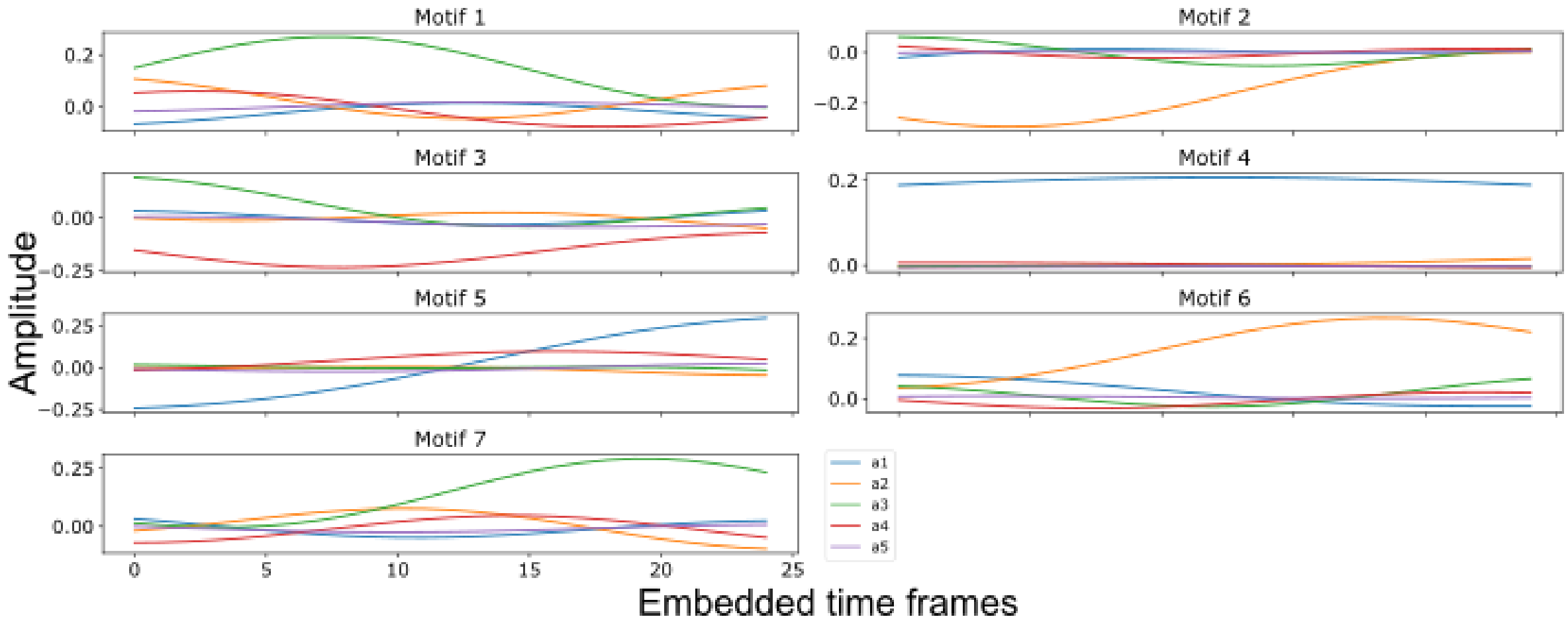


**Fig. S5. Elementary behavioral motifs obtained by TDE-RICA.**

Each panel shows one motif as temporal patterns of 5 eigenworm amplitudes (labeled as a1-a5). The x-axis represents the embedded time within the motif window, and the y-axis represents the motif amplitude.

Biology Open • Supplementary information

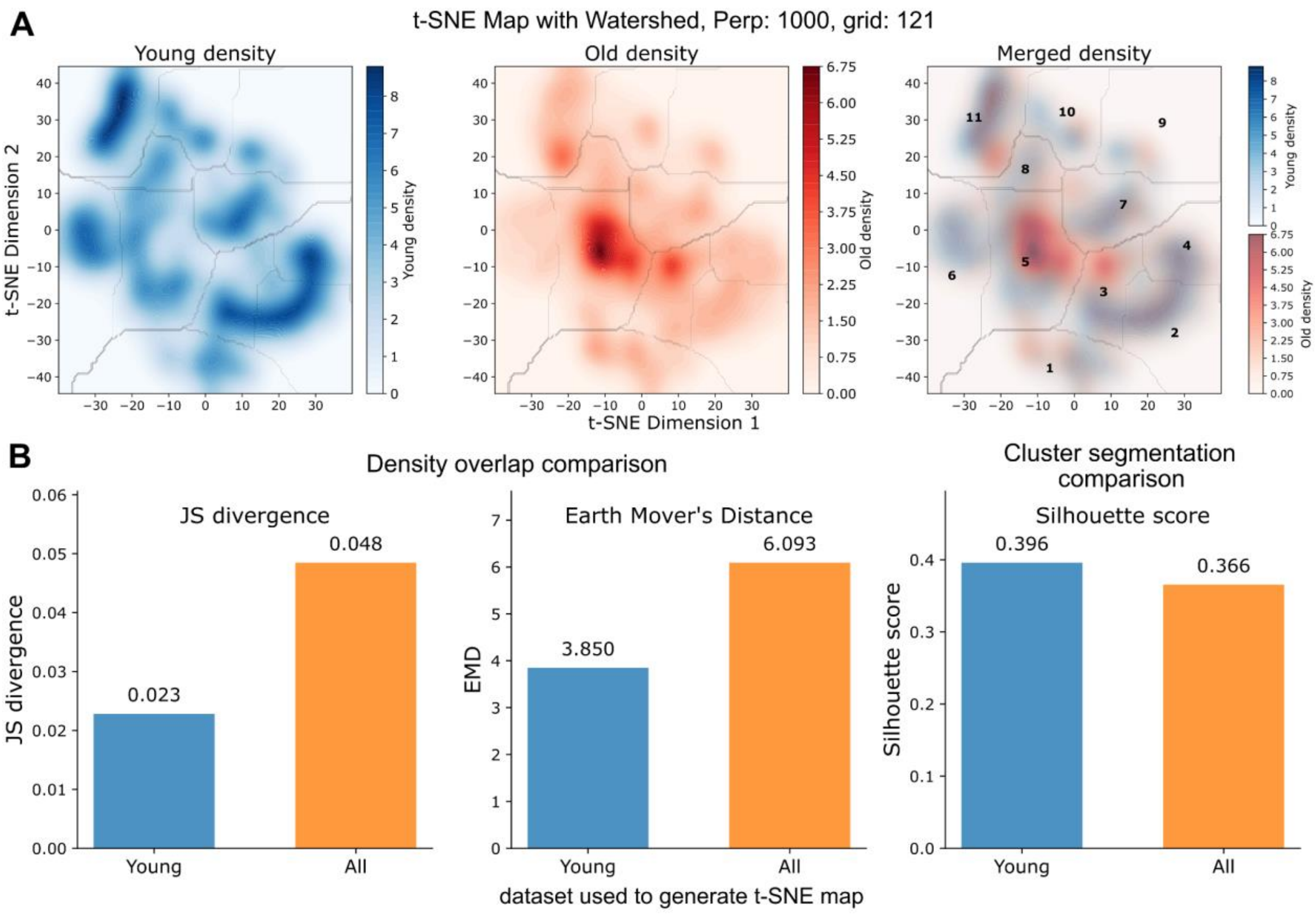


**Fig. S6. Comparison of t-SNE maps generated from young-only and combined datasets** (A) t-SNE map of the probability density generated from all datasets (both young and aged). Cluster segmentation was performed by a watershed algorithm. (B) Comparison of density overlap and cluster segmentation performance between young only and all datasets using Jenson-Shannon (JS) divergence, Earth mover's distance (EMD) and Silhouette score.

Biology Open • Supplementary information

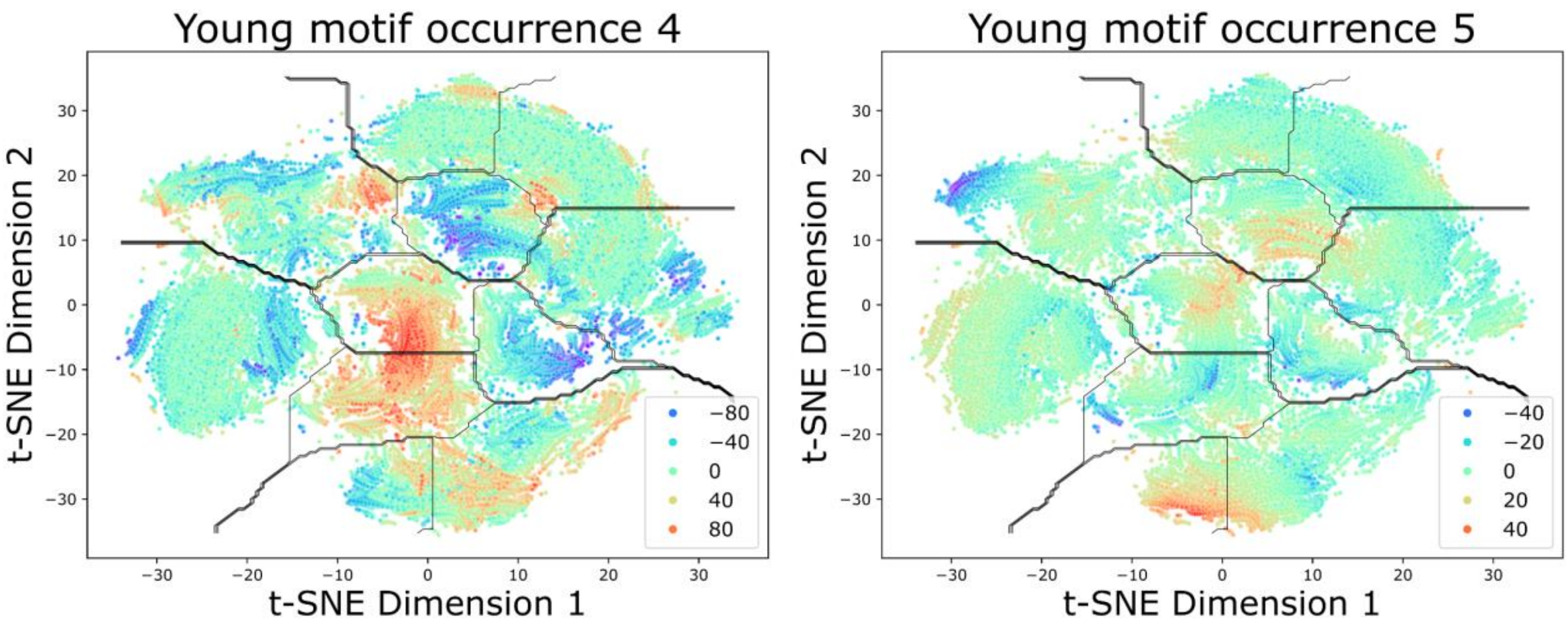


**Fig. S7. t-SNE projection of motif 4 and motif 5 occurrences in young worms.**
Each point represents a frame in the embedded space, and the color intensity indicates the motif occurrence weight.

Biology Open • Supplementary information

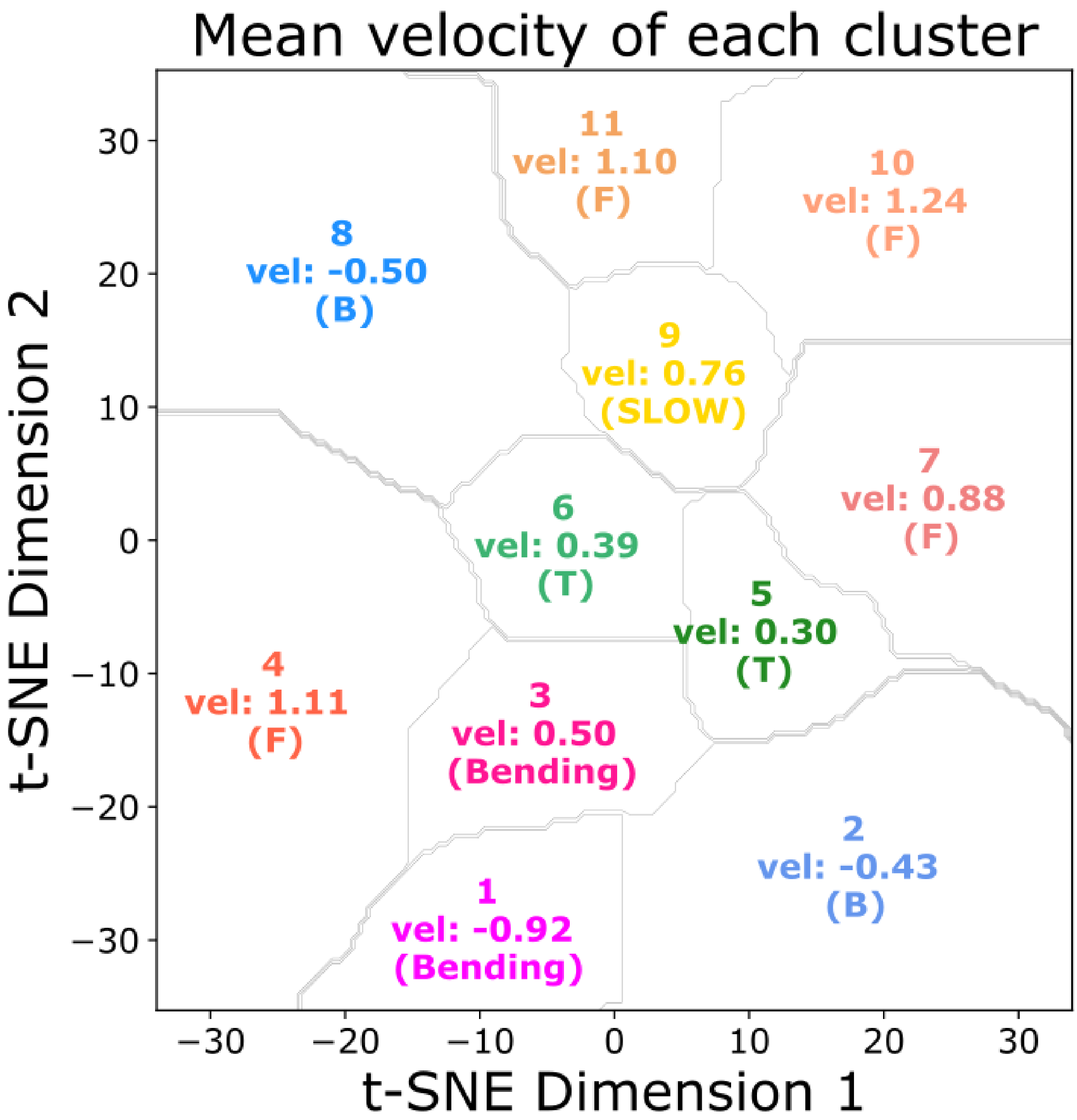


**Fig. S8. t-SNE map of behavioral clusters in young worms.**

Each cluster is shown in a distinct color corresponding to its behavioral state. The mean velocity of each cluster is indicated on the map.

Biology Open • Supplementary information

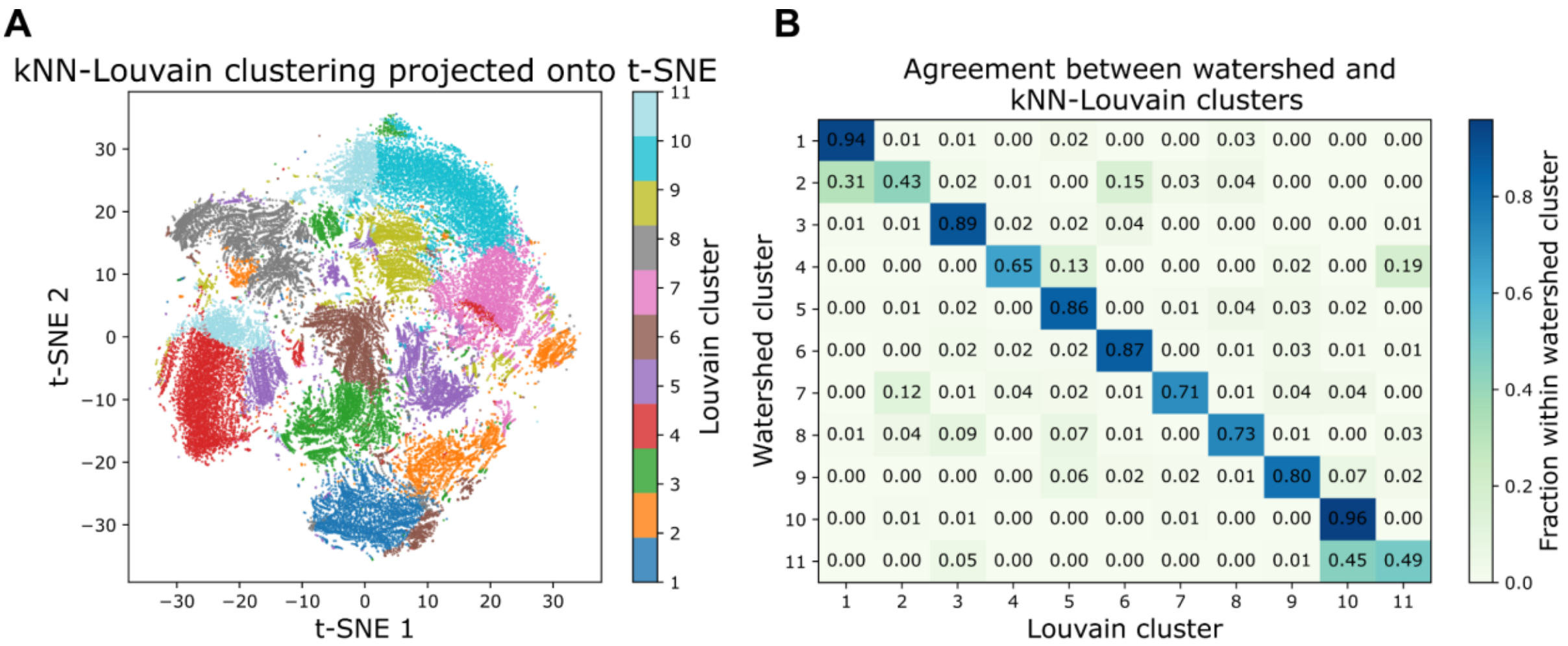


**Fig. S9. Validation of t-SNE-watershed clustering using KNN-Louvain clustering.** (A) KNN-Louvain cluster labels projected onto the t-SNE map. Each dot represents one time point. (B) Agreement between the t-SNE-watershed clusters and the kNN-Louvain clusters. The agreement was quantified using the adjusted Rand index (ARI) and normalized mutual information (NMI).

Biology Open • Supplementary information

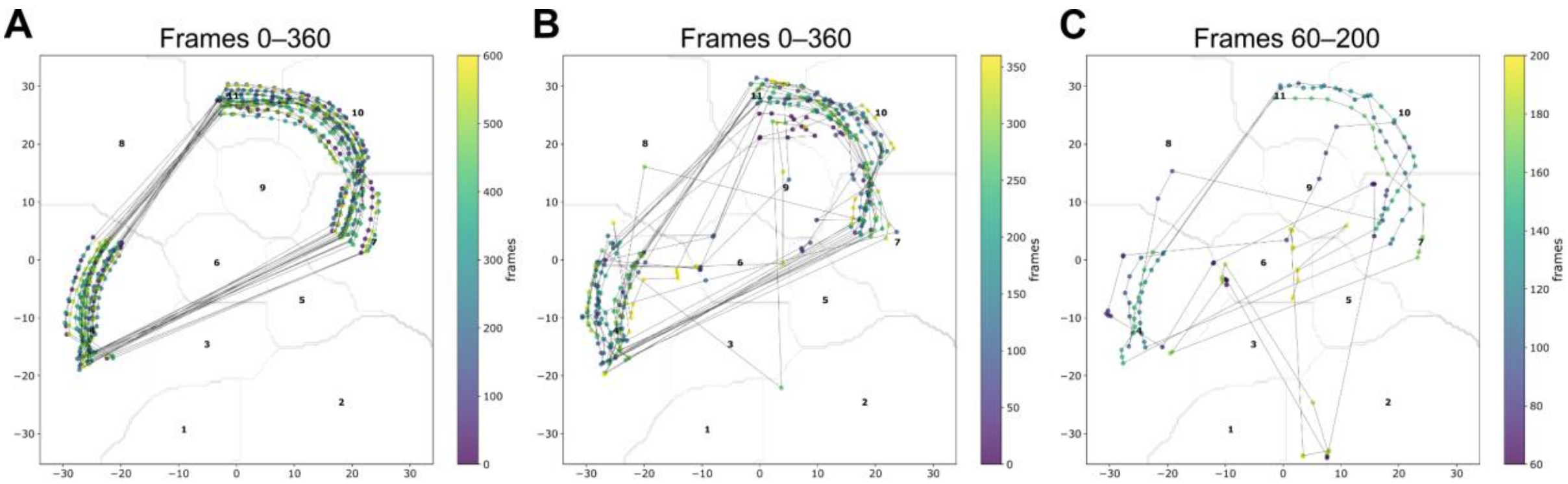


**Fig. S10. Forward movement trajectories on t-SNE map**

(A) One sample each from the young group. (B, C) Representative samples from different aged worms. Colored trajectory corresponds to a continuous time-series movement.

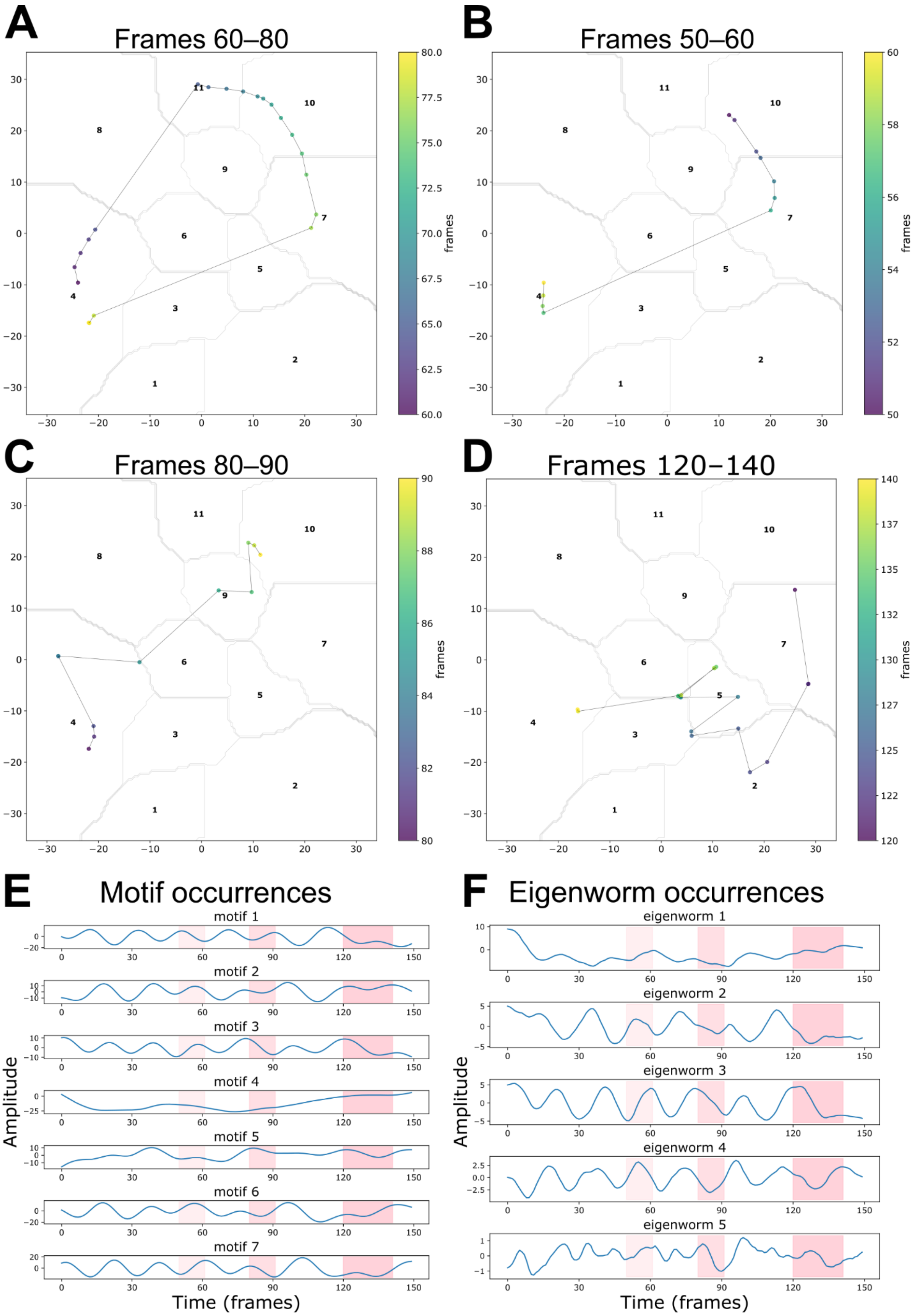


**Fig. S11. Representative smooth and irregular forward trajectories and corresponding motif and eigenworm dynamics.**

Biology Open • Supplementary information

(A-D) Representative smooth and irregular forward movement trajectories projected onto the t-SNE behavioral map. (E, F) Corresponding motif occurrences and eigenworm amplitudes over time. Smooth forward trajectories (A, B) were associated with relatively regular motif and eigenworm dynamics, although the example in panel B showed minor unevenness in the eigenworm amplitude profiles. Irregular forward trajectories (C, D) showed uneven amplitude changes, small bumps, or distorted waveforms, including a loss of clear sinusoidal patterns in the motif and eigenworm amplitudes. Pink shaded regions in E and F indicate the time windows corresponding to the trajectory examples shown in A-D.

Biology Open • Supplementary information

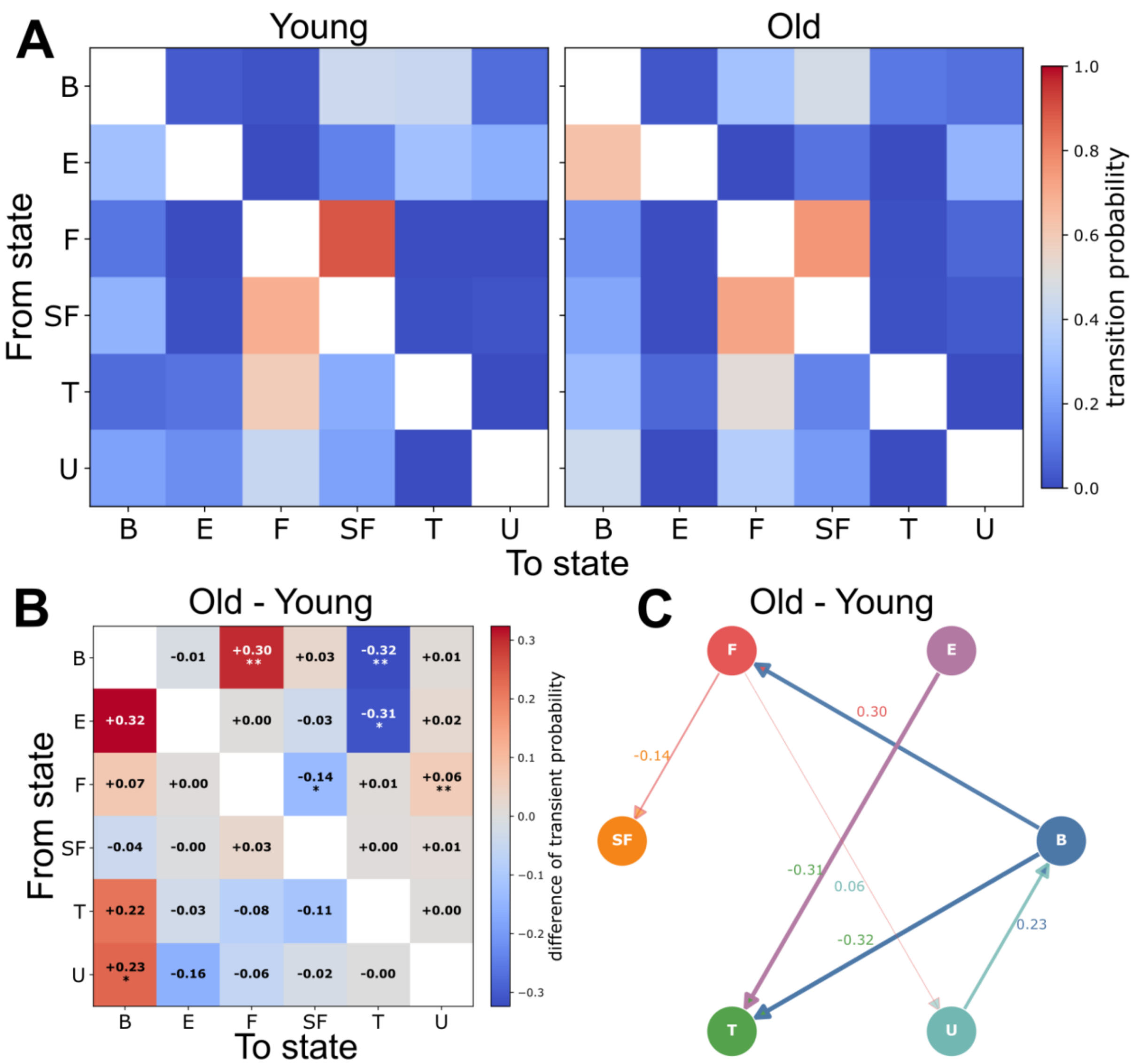


**Fig. S12. Age-related changes in behavioral state transitions in wild-type strain.** (A, B) Heatmaps show the transition probability matrices for the young and aged groups (A), the differences between two groups are plotted as Old - Young (B). Rows indicate the from-state and columns indicate the to-state. Mann-Whitney U test**,** **p* < 0.05, ***p* < 0.01, ****p* < 0.001. The statistical analysis was performed at the level of individual worms. Sample sizes were n = 7 worms for the young group and n = 9 worms for the aged group. (C) Directed network representations of the Old - Young matrix, where nodes denote states and edges represent transitions that differed significantly between the young and aged animals. Edge thickness and transparency reflect transition strength.

Biology Open • Supplementary information

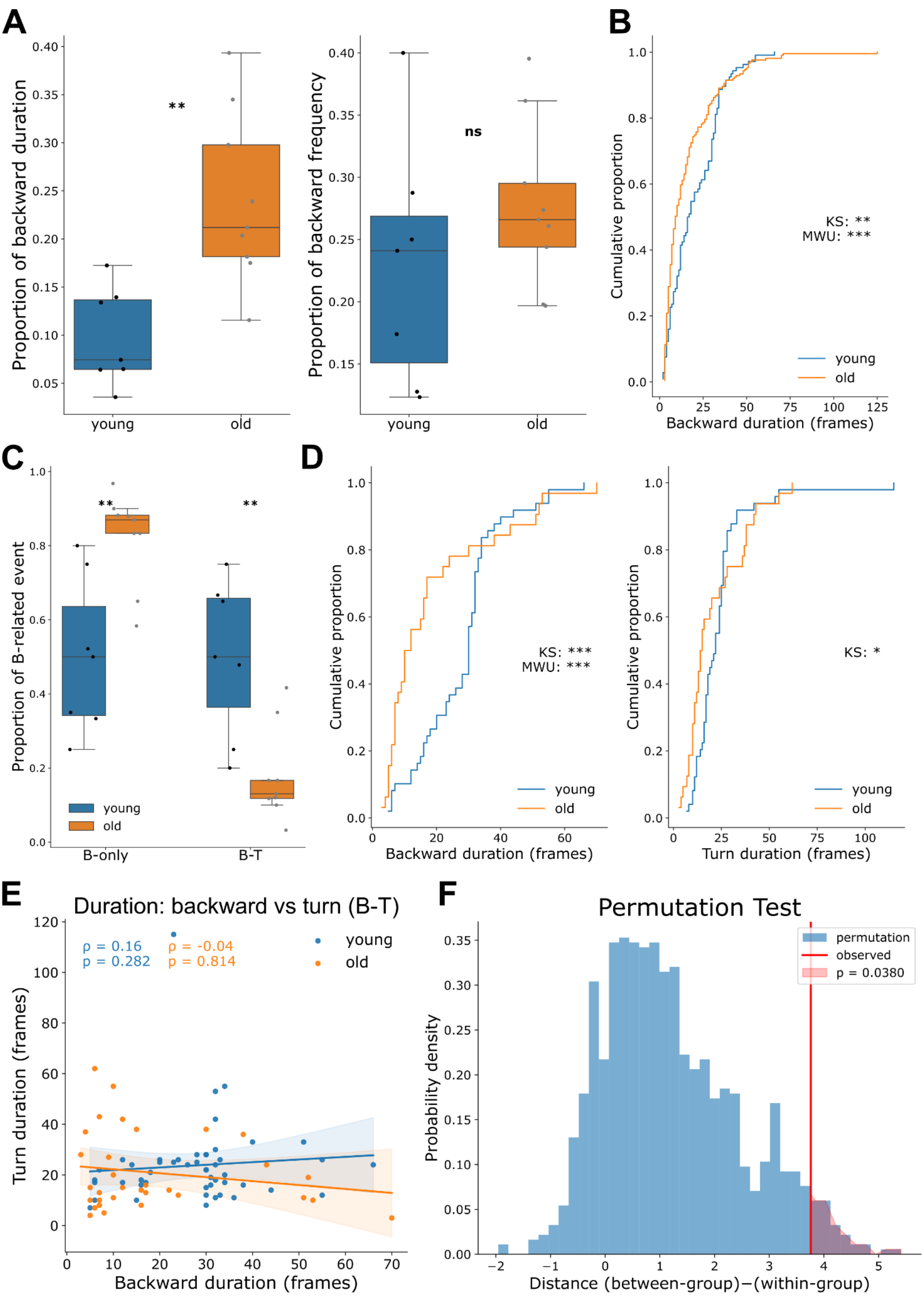


**Fig. S13. Age-related changes in backward and turn related behaviors in wild-type strain.**

(A) Proportions of backward-related events. Fraction of total recording time spent in backward locomotion (Left) and the frequency of backward events (Right). (B) Empirical cumulative

Biology Open • Supplementary information

distribution functions (ECDF) of backward duration. The curves show the cumulative probability of backward durations for young and aged worms. (C) The fraction of B-only and B-T events among all backward events. (D) ECDF of backward duration (left) and turn duration (right) for the B-T events. (E) Relationship between backward and turn duration in B-T event. Each point represents one event pair, and $p$ represents Spearman's rank correlation. (F) Permutation test of DTW-based event chain shape separation between groups. Statistical analysis: For panels A and C, significance was determined using a Mann-Whitney U test; ***$p < 0.001$. Boxes indicate the interquartile range, center lines indicate the median, and whiskers extend to 1.5× the interquartile range. Sample sizes were n = 7 worms for the young group and n = 9 worms for the aged group. And for panels B and D, distribution shapes and central tendencies were evaluated using two-sided Kolmogorov-Smirnov and two-sided Mann-Whitney U tests, respectively; *$p < 0.05$, **$p < 0.01$, ***$p < 0.001$. For panel F, group separation of event chain shapes was assessed via a permutation test (1,000 iterations); the observed value (red line) represents the difference between the mean between-group and within-group DTW distances against the permuted null distribution (histogram), $p < 0.05$.

Biology Open • Supplementary information

Biology Open (2026): doi:10.1242/bio.062711: Supplementary information

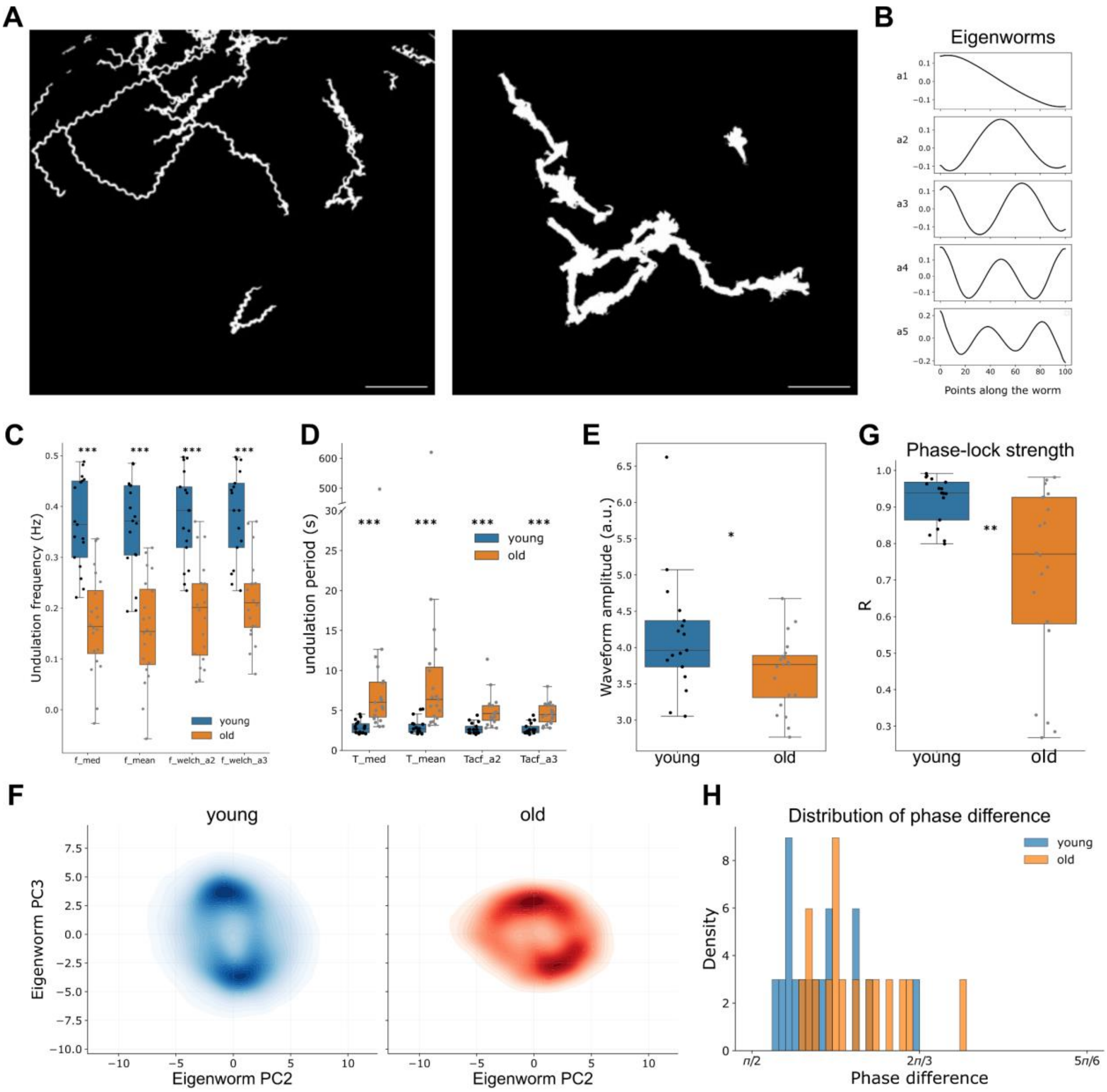


**Fig. S14. Forward locomotion differences in wild-type strain.**

(A) Five-minute locomotion trajectories. Locomotion trajectories were recorded for 5 min from 2 individuals in the young group and 5 individuals in the aged group. (B) Five eigenworms. The x-axis represents body position and the y-axis represents the amplitude of each eigenworm. (C-E, and G) Each dot represents the estimated value from an individual worm, Mann-Whitney U test, $*p < 0.05$, $**p < 0.01$, $***p < 0.001$. Boxes indicate the interquartile range, center lines indicate the median, and whiskers extend to 1.5× the interquartile range. The statistical analysis was performed at the level of individual worms. Sample sizes were n = 7 worms for the young group and n = 9 worms for the aged group. (C) Instantaneous undulation frequency is estimated by multiple methods. Phase based estimates were obtained from the eigenworm phase dynamics and summarized as the median (f_med) or mean (f_mean) values. Dominant undulation frequencies from the peak of the Welch power spectrum of a2 (f_welch_a2) or a3 (f_welch_a3) are also compared. (D) Corresponding undulation periods are estimated by multiple methods. Phase based period estimates were calculated from the instantaneous undulation frequency and summarized as the median (T_med) or mean (T_mean). Dominant period estimated from the first non-zero-lag peak of autocorrelation of a2 (Tacf_a2) or a3

Biology Open • Supplementary information

(Tacf_a3). (E) Amplitude of sinusoidal waveform during forward movement. (F) Eigenworm state-space density. (G) Phase locking between a2 and a3 during forward locomotion. This measures the consistency of the phase relationship between two signals. (H) Histogram of the eigenworm phase offset. This measures the average angle of the phase difference over time.

Biology Open • Supplementary information

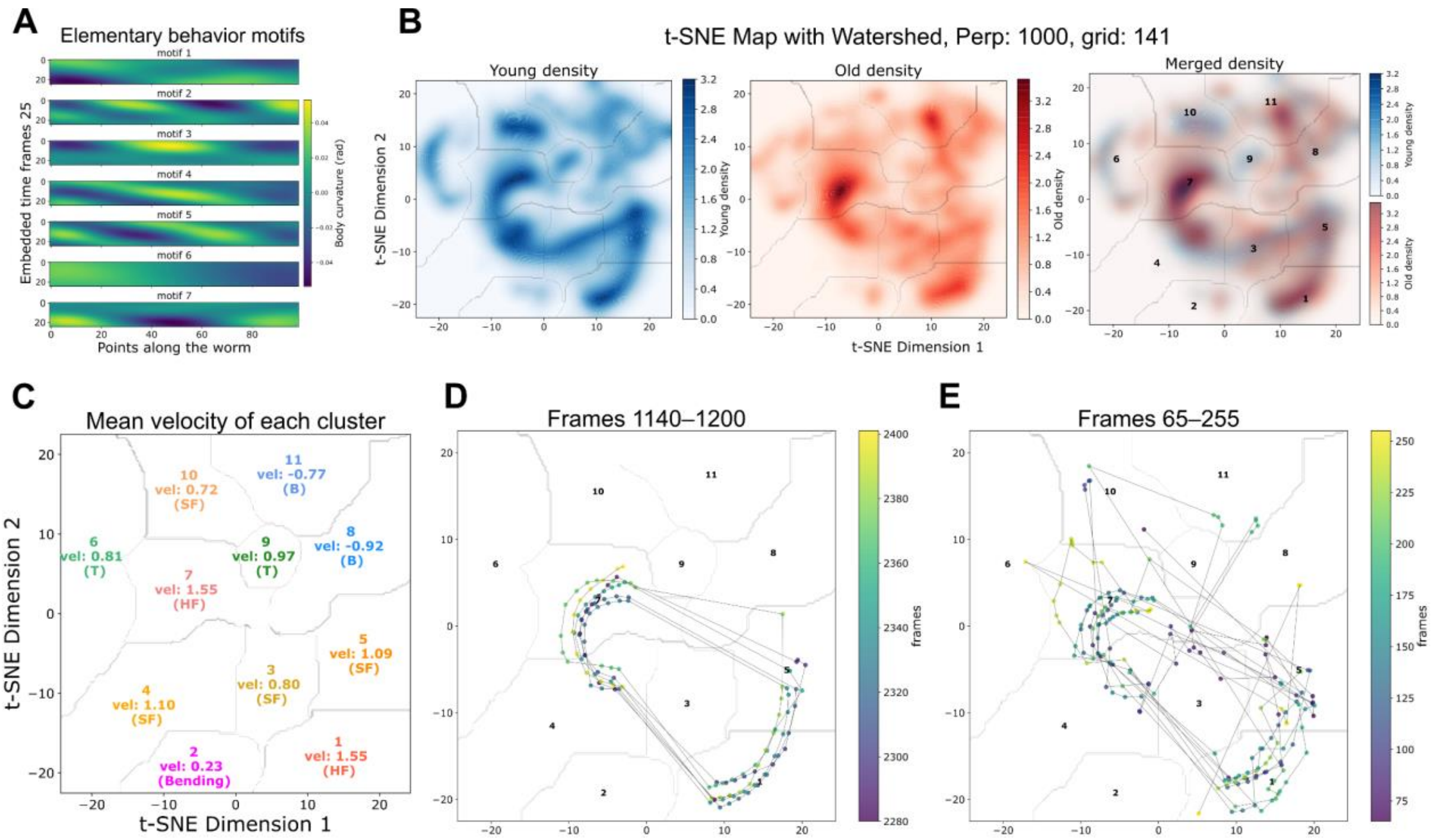


**Fig. S15. Locomotion behavior classification of wild-type strain based on t-SNE and watershed clustering.**

(A) Elementary behavior motifs. The x-axis represents the body position and the y-axis represents the 25 embedded frames. (B) t-SNE probability density of behavioral motif vectors. Clusters were segmented by a watershed algorithm. (C) t-SNE map of behavioral clusters in young worms. Each cluster is shown in a distinct color corresponding to its behavioral state. The mean velocity of each cluster is indicated on the map. (D, E) Forward movement trajectories on t-SNE map from the young (D) and aged (E) group. Colored trajectory corresponds to a continuous time-series movement. Each panel shows one representative sample, with the corresponding time frames indicated in the panel title.

**Table S1.** List of the numbers of backward- and turn-related behavioral events

Available for download at
https://journals.biologists.com/bio/article-lookup/doi/10.1242/bio.062711#supplementary-data

Biology Open • Supplementary information

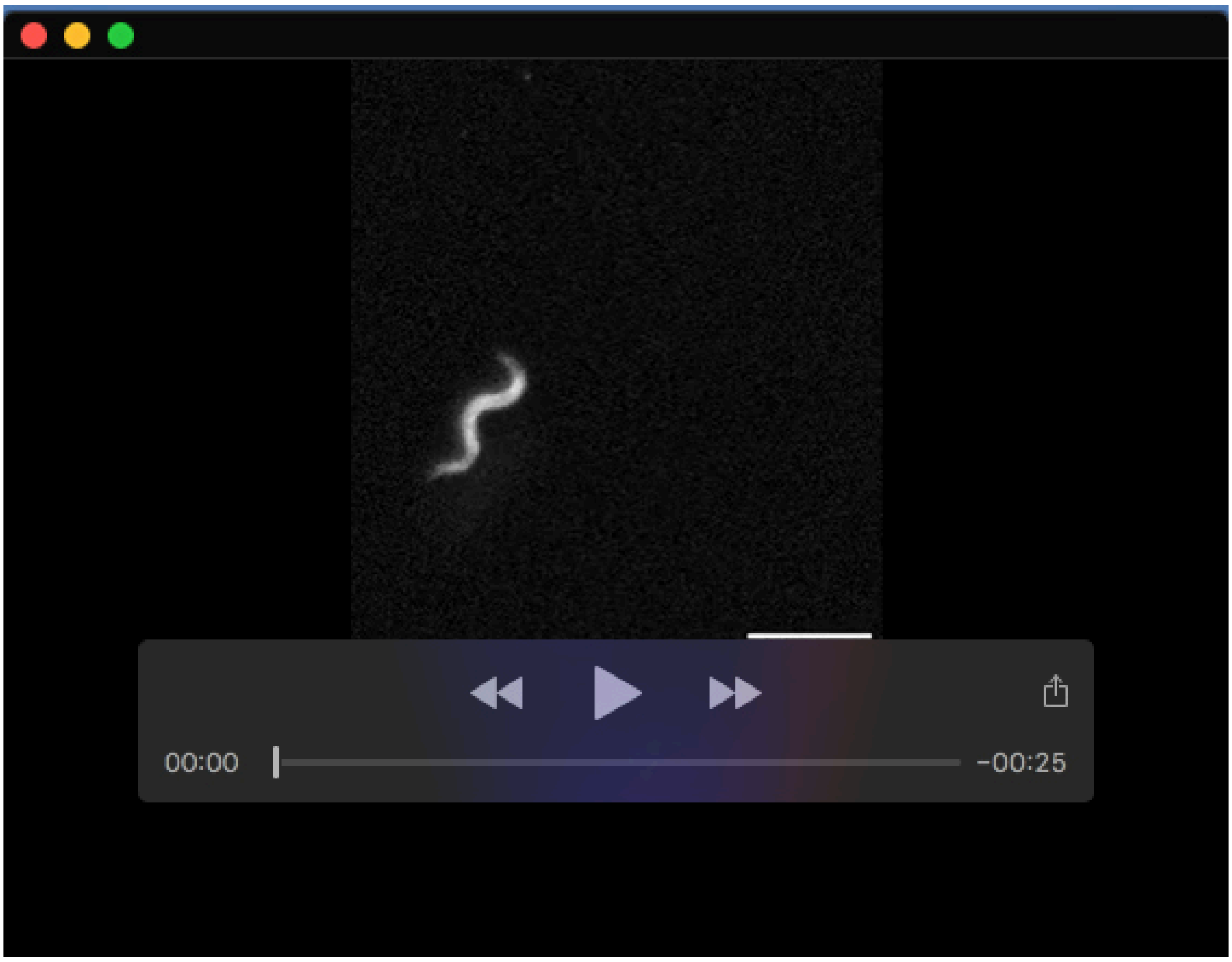


**Movie 1.** Video of a young animal which crawled forward in ~30 s.

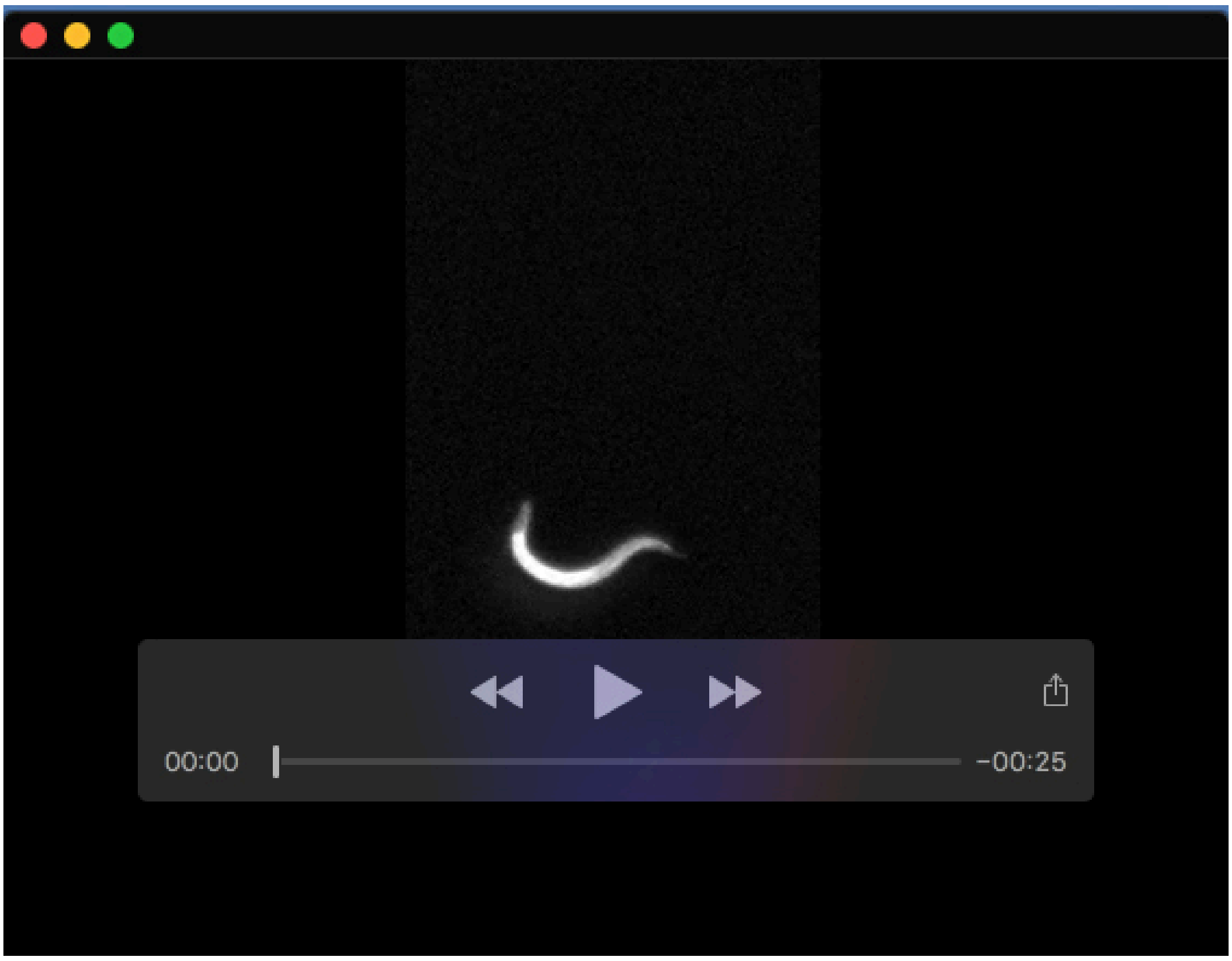


**Movie 2.** Video of an aged animal which crawled forward in ~20 s.

Biology Open • Supplementary information

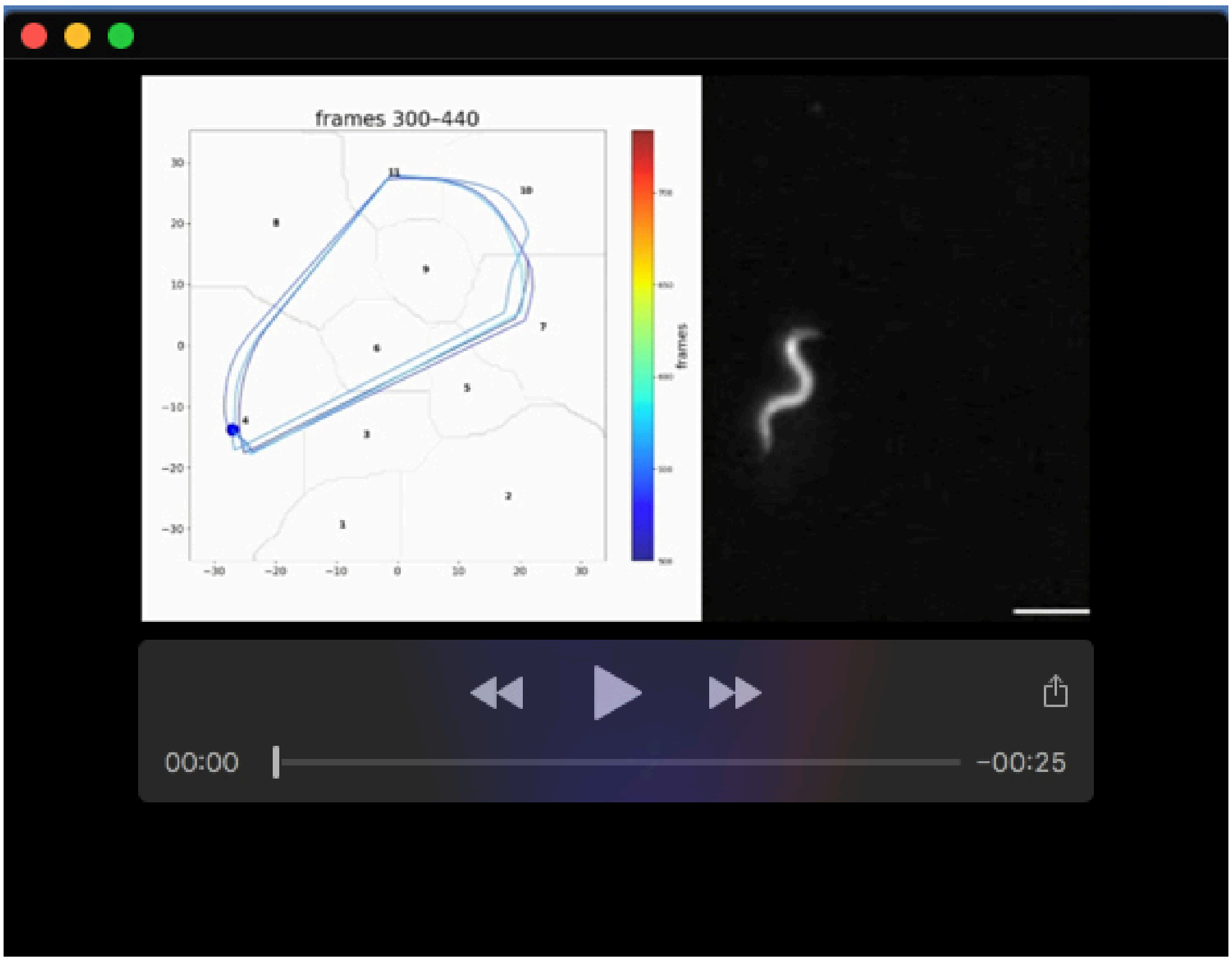


**Movie 3.** Trajectories of forward behavior plotted onto t-SNE map (left). Real movie of forward behavior (right).

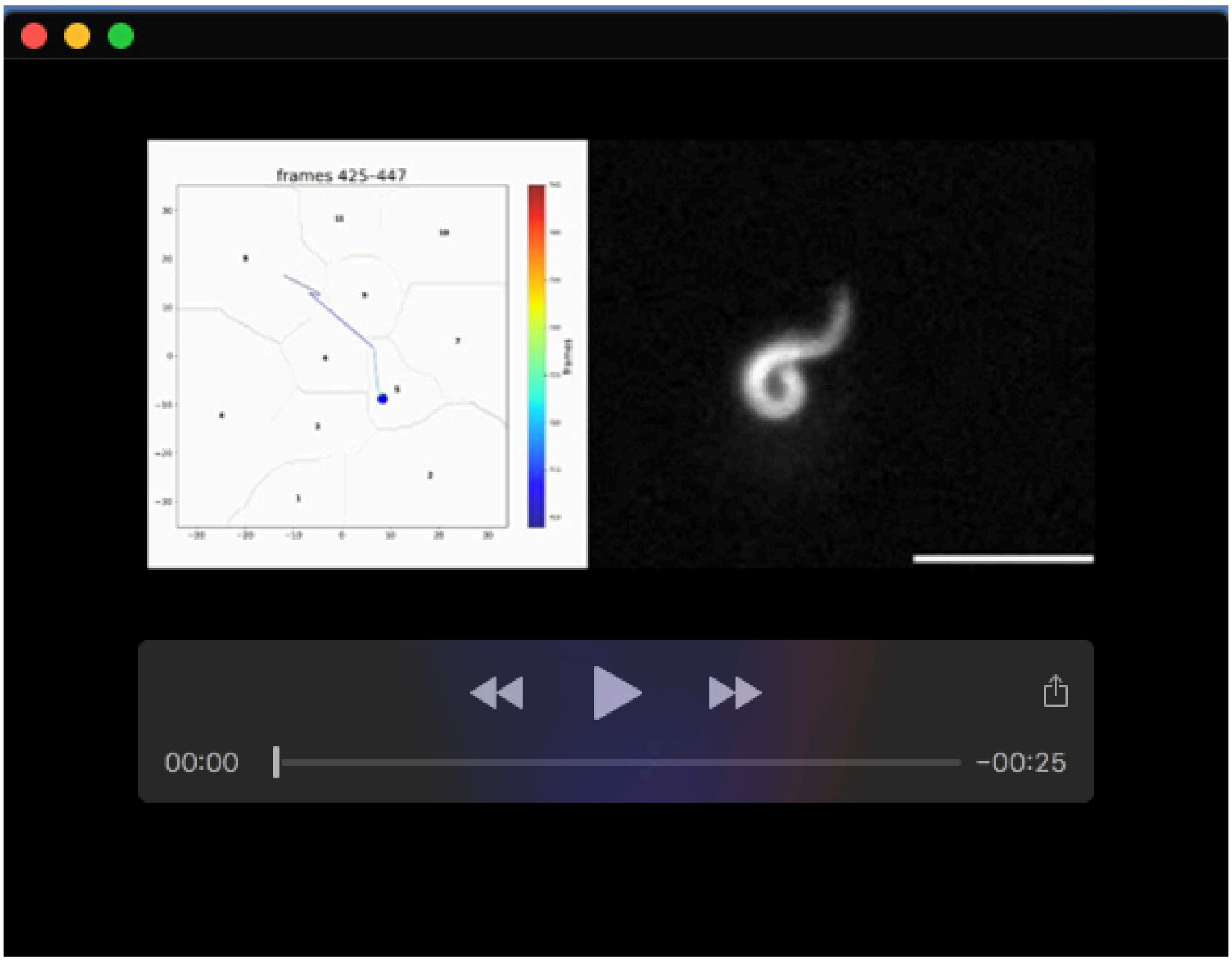


**Movie 4.** Trajectories of backward-turn behavior plotted onto t-SNE map (left). Real movie of backward-turn behavior (right).

Biology Open • Supplementary information

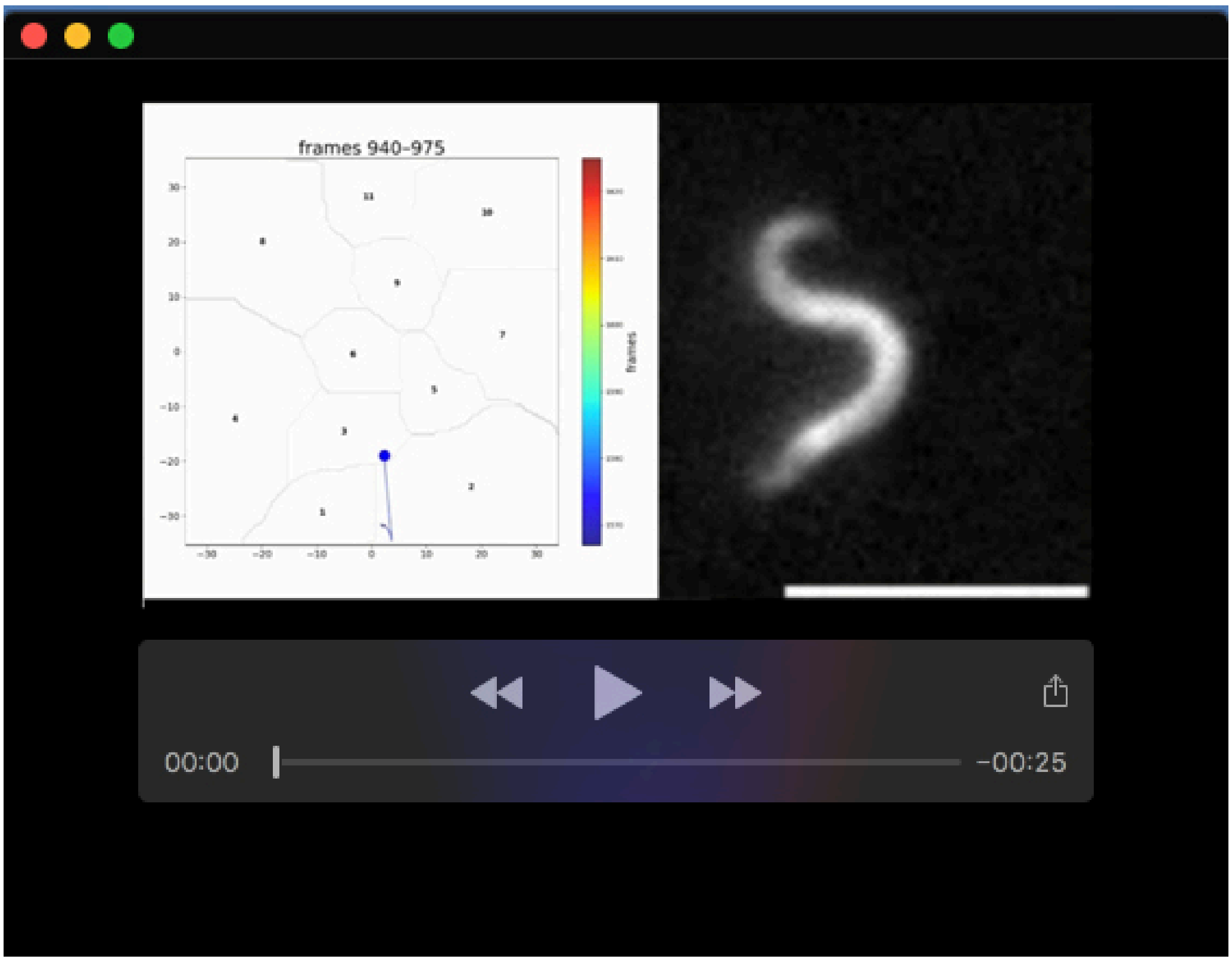


**Movie 5.** Trajectories of backward-turn-bending behavior plotted onto t-SNE map (left). Real movie of backward-turn-bending behavior (right).

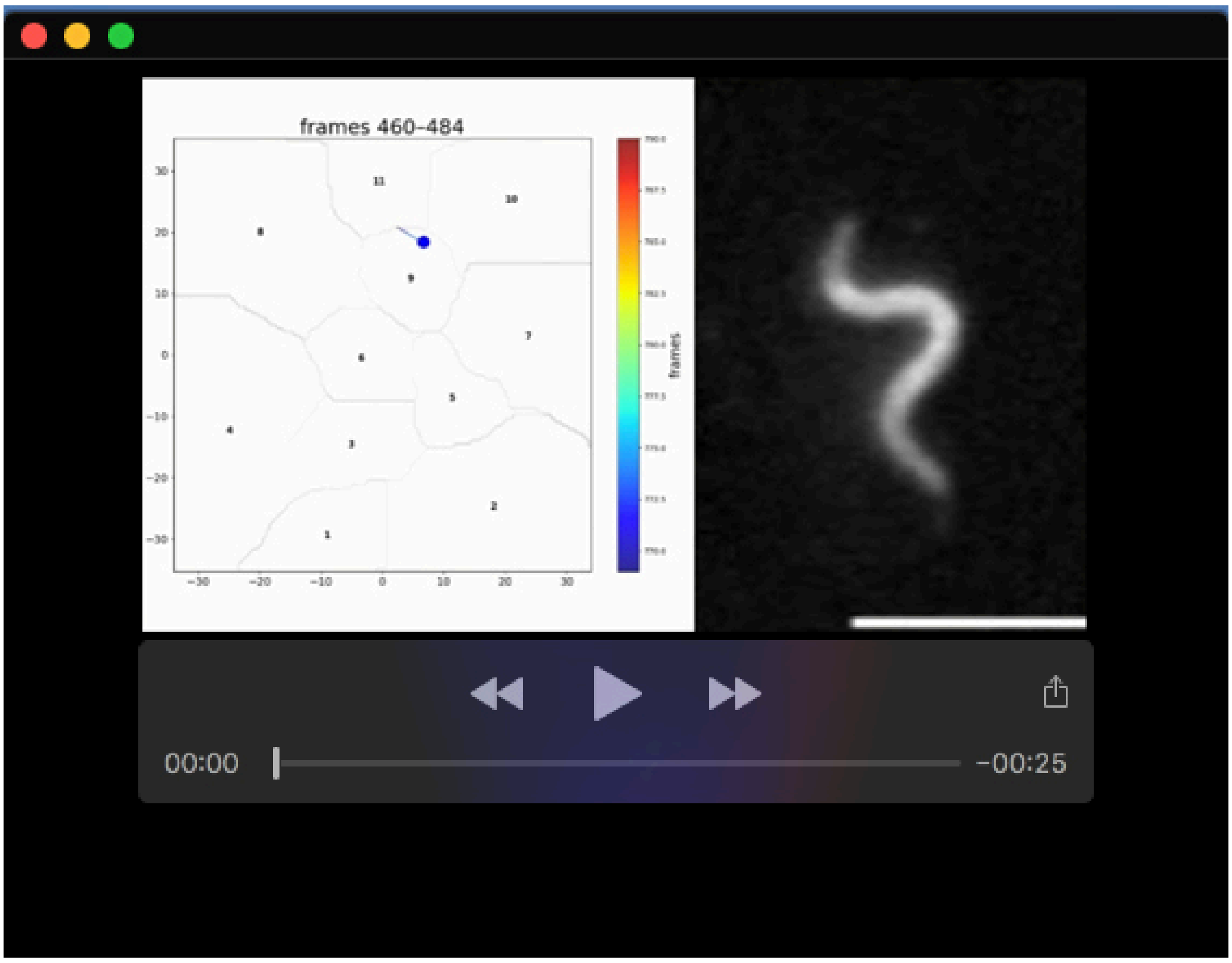


**Movie 6.** Trajectories of slow forward-forward behavior plotted onto t-SNE map (left). Real movie of slow forward-forward behavior (right).

Biology Open • Supplementary information

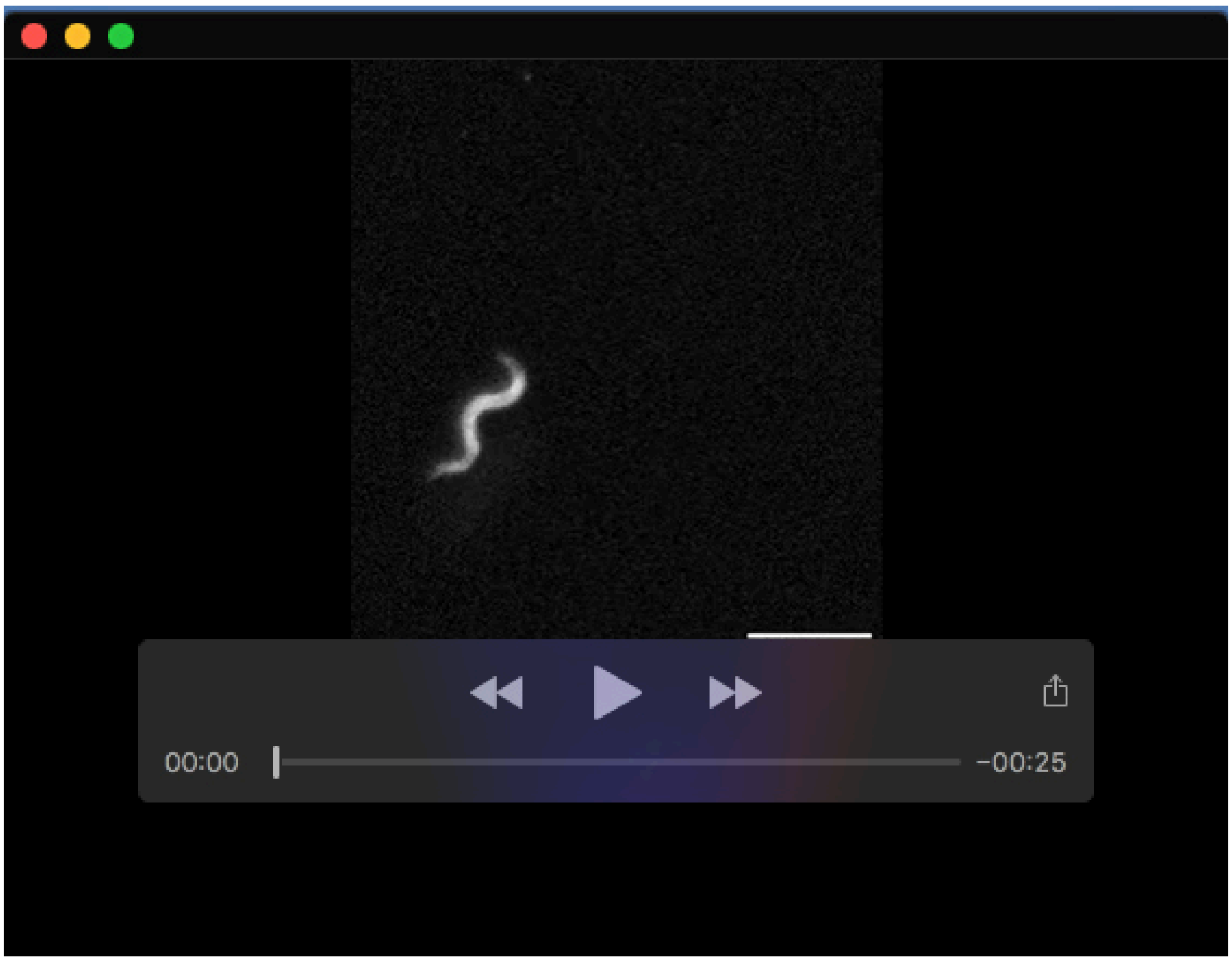


**Movie 7.** Animation of forward movement trajectories from a young animal (The same sample of Movie S1.).

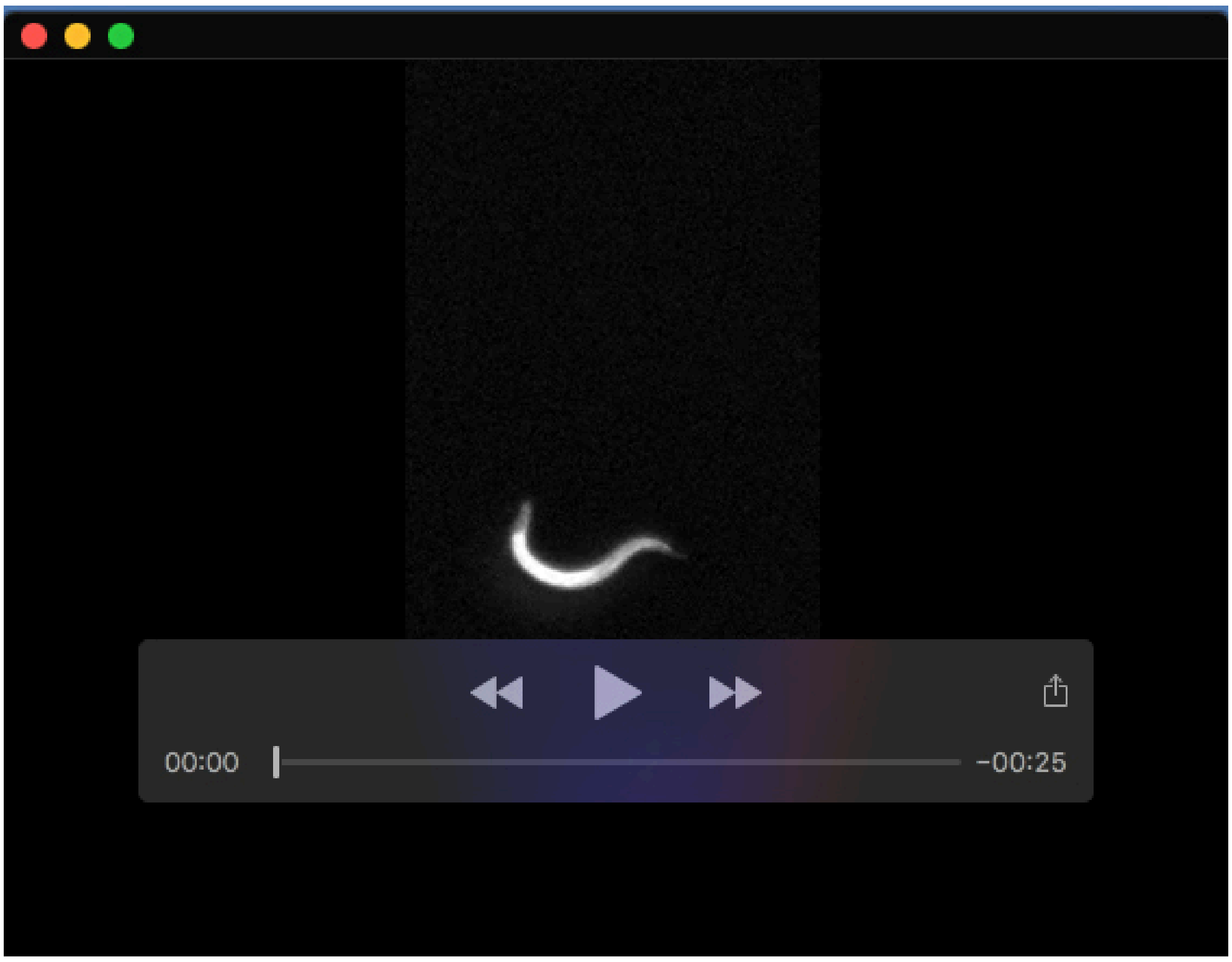


**Movie 8.** Animation of forward movement trajectories from an aged animal (The same sample of Movie S2.).

Biology Open • Supplementary information

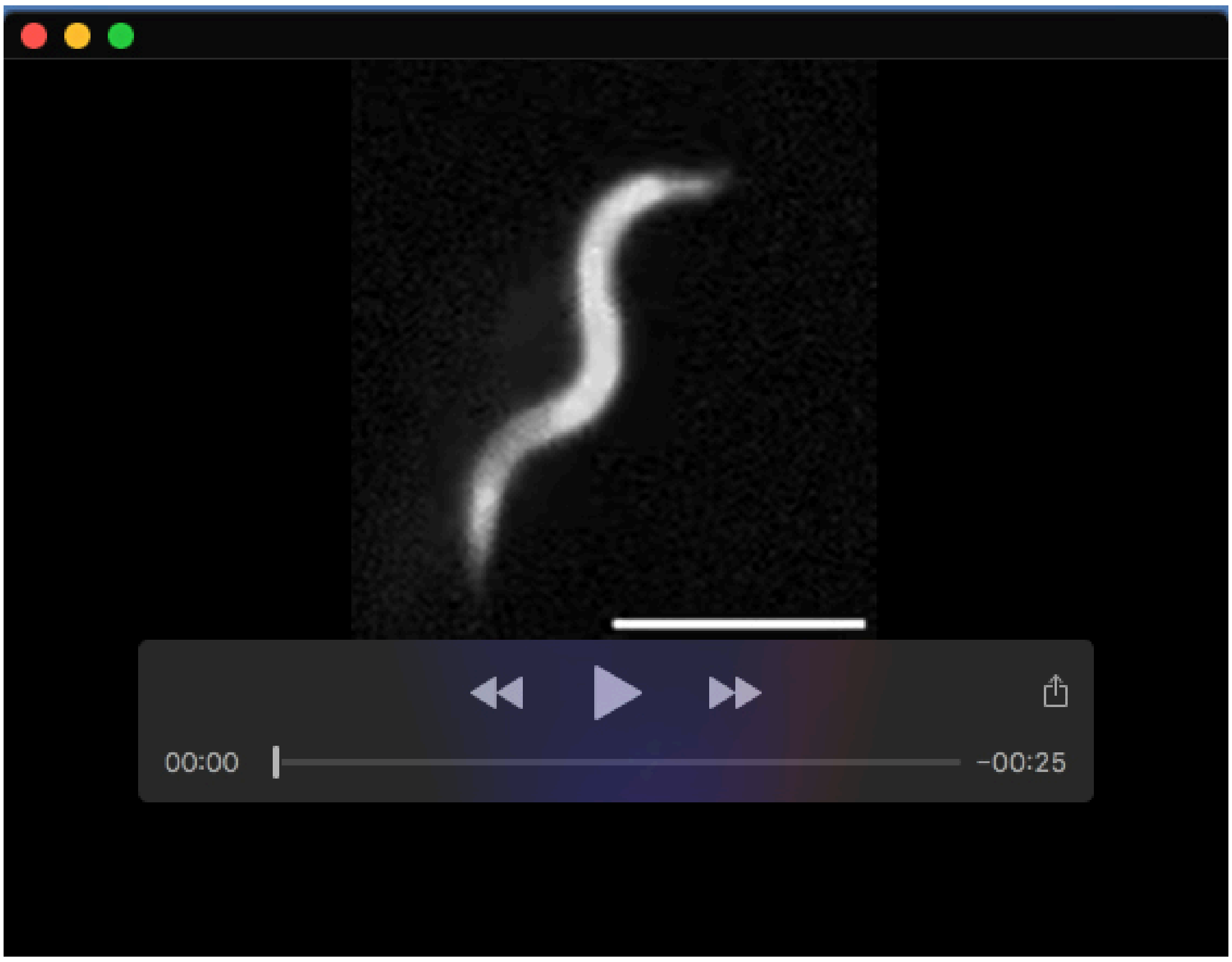


**Movie 9.** Video of an aged animal that crawled forward smoothly at some time points.

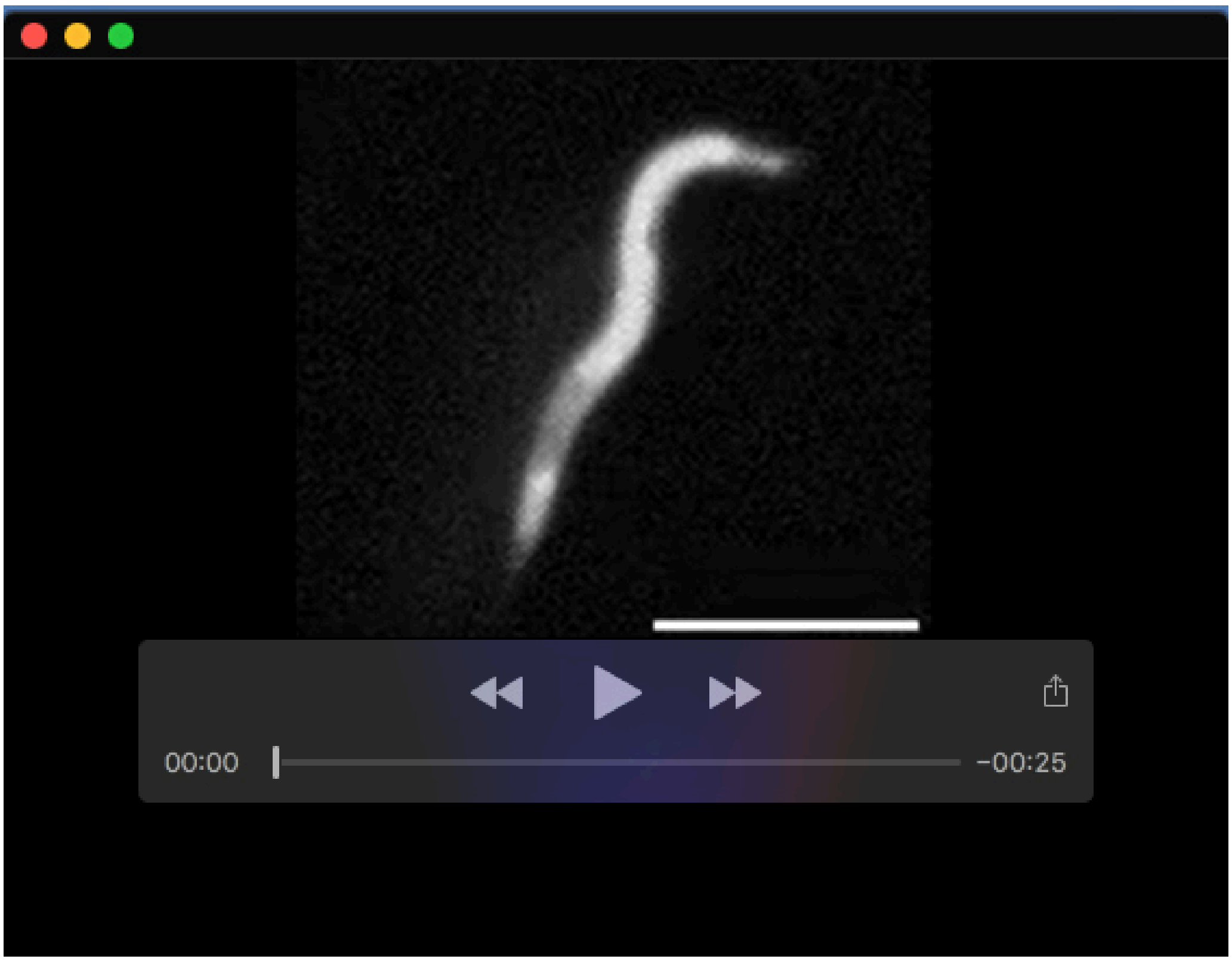


**Movie 10.** Video from the same animal as in Movie S9, showing abnormal forward crawling at some time points.

Biology Open • Supplementary information

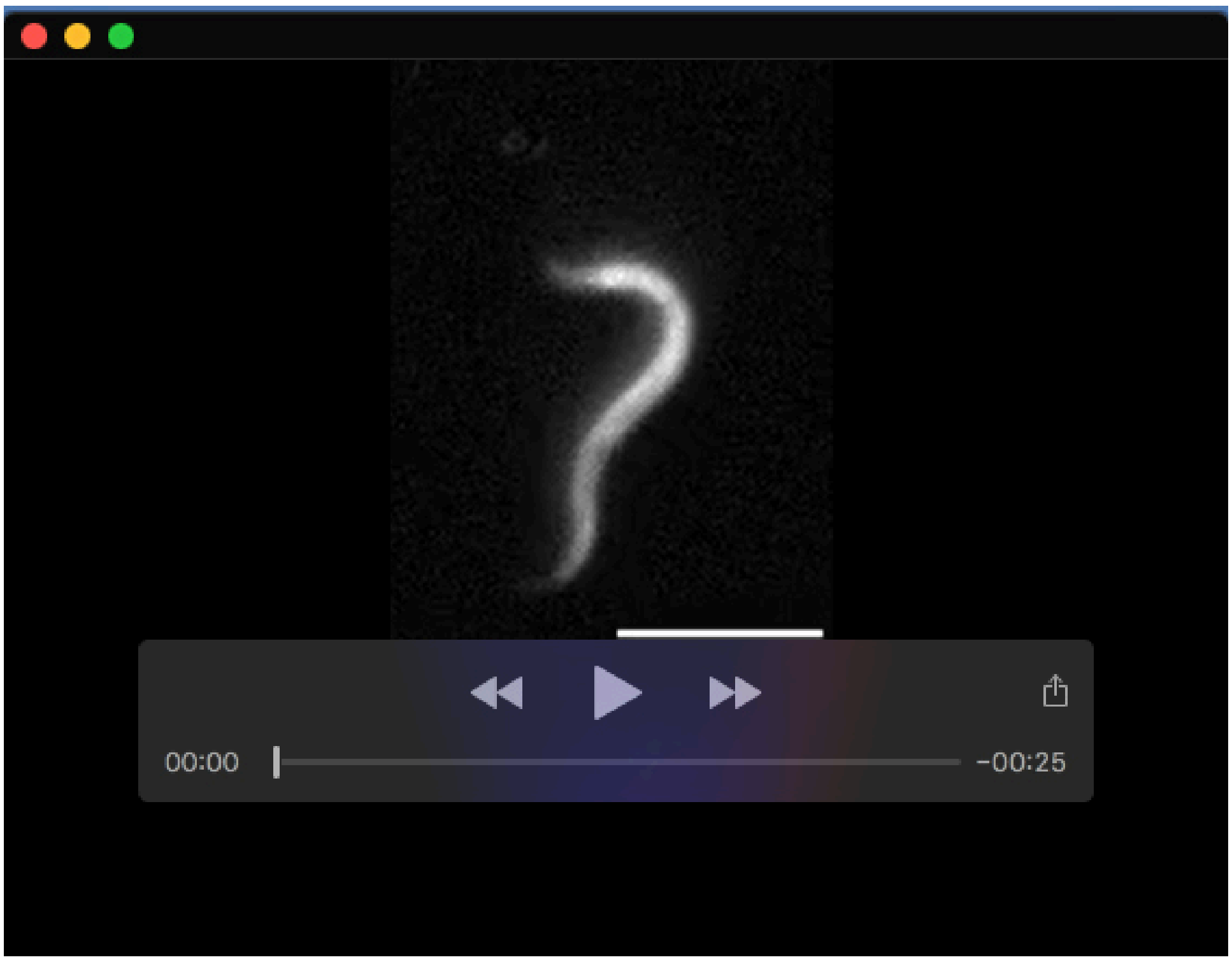


**Movie 11.** Video of another aged animal that crawled forward smoothly at some time points.

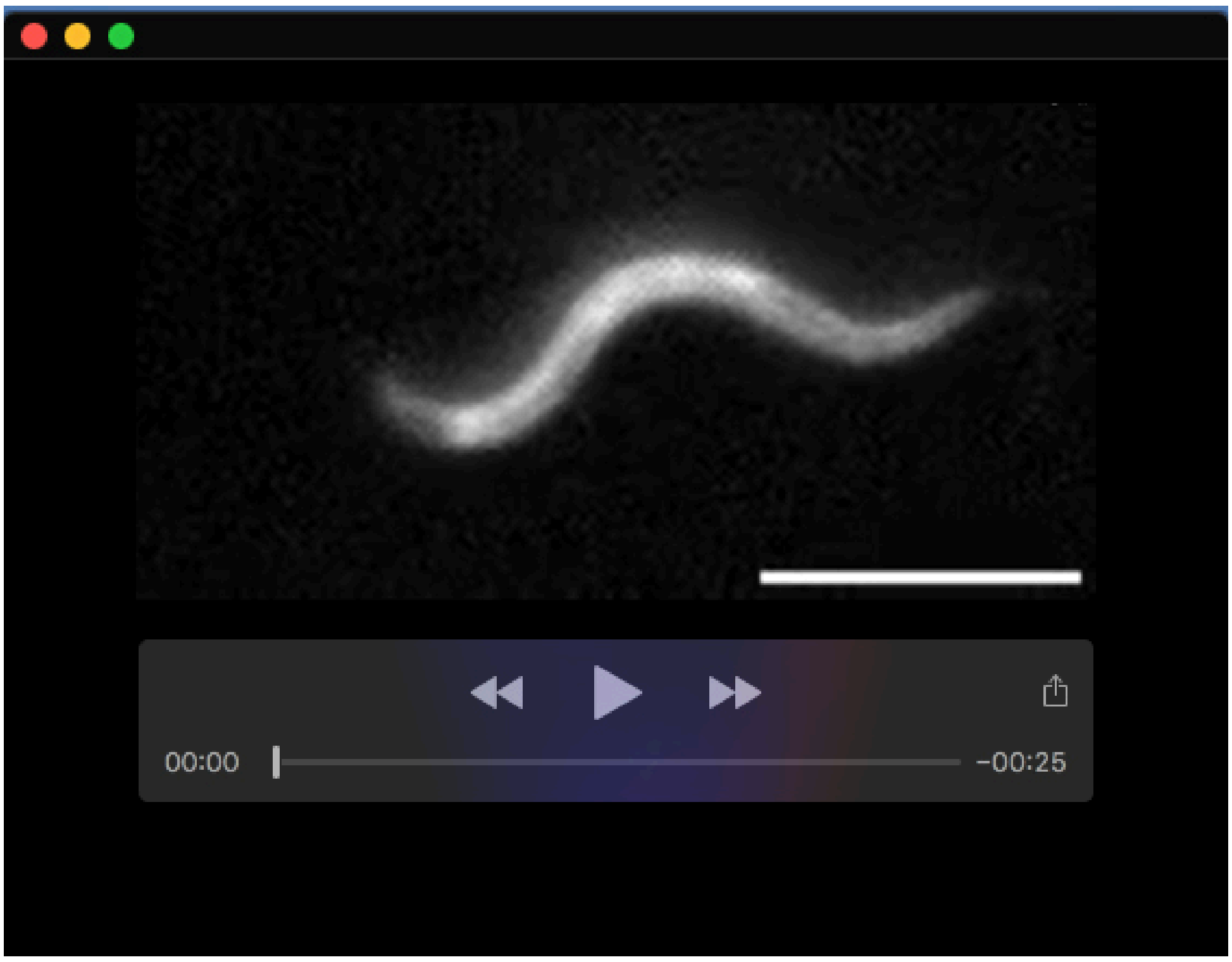


**Movie 12.** Video from the same animal as in Movie S11, showing abnormal forward crawling at some time points.
