# Supplementary material for: Aging-related behavioral defects in Caenorhabditis elegans
Source: Biol Open. 2026 Aug 17;15(8):bio062711. doi: 10.1242/bio.062711 (PMC13552477; doi:10.1242/bio.062711)
Supplement: Supplementary file 2 [file biolopen-15-062711_review_history.pdf]

Aging related behavioral defects in C. elegans

Jingxuan Zeng, Chung-Kuan Chen, Yuichi Iino and Yu Toyoshima
10.1242/bio.062711

Editor: Sandhya Koushika

Review timeline

Original submission:	22 May 2026
Editorial decision:	1 June 2026
First revision received:	23 June 2026
Accepted:	23 July 2026

Original submission

First decision letter

MS ID#: bio.062711

MS Title: Aging related behavioral defects in C. elegans

Authors: Jingxuan Zeng, Chung-Kuan Chen, Yuichi Iino and Yu Toyoshima

I have now reached a decision on the above manuscript.

The reviewer reports are shown at the bottom of this email.

As you will see, the reviewers raised a number of substantial criticisms that prevent me from accepting the paper at this stage.

They suggest, however, that a revised version might prove acceptable, if you can address their concerns. If you think that you can deal satisfactorily with the criticisms on revision, I would be pleased to see a revised manuscript. We would then return it to the reviewers.

At this stage, we also ask you to ensure your manuscript complies with our formatting guidelines. Provided you are able to fully address the referees' comments, we are positive about publication of your paper (we accept over 95% of revision submissions) and therefore hope you won't mind any extra work involved in reformatting your manuscript at this point.

Please upload both a 'clean' version of your Word file, along with a highlighted version clearly showing where you have made changes in the revised manuscript. Please avoid using 'Track changes' in Word files as these are lost in PDF conversion.
I should be grateful if you would also provide a point-by-point response detailing how you have dealt with the points raised by the reviewers in the 'Response to Reviewers' box. Please attend to all of the reviewers' comments. If you do not agree with any of their criticisms or suggestions please explain clearly why this is so.

Reviewer 1

Comments for the author

© 2026. Published by The Company of Biologists under the terms of the Creative Commons Attribution License (https://creativecommons.org/licenses/by/4.0/).

Zeng et al. investigate age-associated changes in locomotor behavior using a quantitative behavioral analysis framework in C. elegans. The authors compare young (day 1) and aged (day 9) animals and report a series of aging-associated alterations in locomotor behavior.
Although the manuscript addresses an important biological question and presents a potentially valuable dataset, several aspects of the experimental design and data analysis require substantial clarification. In addition, further validation is needed to support the conclusions and improve the presentation of the data in both the figures and their legends. The manuscript would also benefit from a more balanced discussion of the biological significance of the findings, as well as their limitations and broader implications.
Below are listed the points to address.

Major comments
1. The number of worms analyzed in each group, day 1 and day 9, is not reported in the manuscript, figures, figure legends, or Methods section. It is also unclear whether the experiments were performed using a single batch of animals, corresponding to one biological replicate, or whether multiple independent batches were analyzed. This information is essential for evaluating the robustness of the results and the appropriateness of the statistical analyses. The authors should clearly report the number of animals, the number of independent biological replicates, and how these were incorporated into the statistical analysis. Ideally, the experiments should be performed across multiple independent batches, or alternatively the authors should provide a power analysis justifying the sample size used.
2. The information regarding the strain used in the study requires clarification. In particular, the rationale for using the TOY21 strain should be explained, including whether this strain carries genetic modifications that could influence locomotor behavior or aging-related phenotypes. To strengthen the generality of the conclusions, the key findings should also be validated in a standard wild-type strain.
3. In Figure 2A, the authors present the analytical pipeline. However, the schematic representation of the different analytical steps is relatively complex and difficult to follow. The authors should provide additional clarification in the figure legend and/or simplify the schematic. Alternatively, this panel may be more suitable as a dedicated supplementary figure, with the main text focusing on the key conceptual steps of the analysis.
4. In Figure 2C, the authors present a clustering analysis based on the generation of a density map in a two-dimensional projection of the multidimensional t-SNE space, followed by watershed-based cluster identification. Two major concerns arise from this approach. First, the method is not sufficiently justified and appears unconventional for this type of analysis, as clustering is performed on a two-dimensional projection rather than in the original multidimensional feature space. More standard unsupervised clustering approaches, such as k-nearest-neighbor-based clustering, Louvain/Leiden clustering, or other methods applied before or independently of dimensionality reduction, would be more appropriate and less dependent on the distortions introduced by t-SNE visualization. Second, the cluster boundaries shown in the figure do not appear to match the density distribution of the data particularly well and do not seem to capture the organization of the young and aged worm datasets in a convincing way. The authors should therefore repeat or validate the clustering analysis using dimensionality reduction methods such as t-SNE or UMAP coupled with clustering approaches that are not based solely on two-dimensional projections. This would help determine whether the identified behavioral clusters are robust and not an artefact of the visualization method.
5. In Figure 2C, the authors indicate the presence of 11 clusters. However, in Figure 2D the clusters are labeled from 0 to 11, which corresponds to 12 clusters. In addition, "cluster 12" is mentioned in the text at line 162. The authors should carefully revise this section and ensure consistency among the text, figures, labels, and analysis.
6. In Figures 2E-G, it is unclear how many worms were included in the analysis. The figure legends do not provide sufficient information to understand the sample size, the number of biological replicates, or how the data were aggregated. The authors should clarify these points in the legends and Methods section.
7. In Figures 3A-B, the subtraction matrix appears to provide only a descriptive comparison between groups, without statistical testing to support the reported differences. The authors should include appropriate statistical analyses to assess whether the observed changes in transition probabilities are significant. For example, they could apply a chi-square test, or calculate transition probabilities for each individual worm and then perform statistical testing across biological replicates. This would provide stronger support for the conclusions drawn from these data.

© 2026. Published by The Company of Biologists under the terms of the Creative Commons Attribution License (https://creativecommons.org/licenses/by/4.0/).

8. In Figure 5, panels A and B appear to be switched. The descriptions in the text and figure legend do not match the corresponding panels. The authors should correct the figure, legend, and text to ensure consistency.
9. In Figure 5, it is unclear whether the calculations are based on the t-SNE analysis shown in Figure 2. This information is missing from both the main text and the figure legend. The authors should clearly explain how these calculations were performed and whether they derive from the same dimensionality-reduction space used in Figure 2.
10. In the Discussion section, the authors should provide appropriate references for all major claims, particularly in the section comparing worm locomotor aging with human aging. In addition, lines 405-408 appear to contain repetitive statements and should be revised for clarity. The Discussion would also benefit from a more explicit consideration of the limitations of the current study, including limitations related to the experimental design, sample size, behavioral classification approach, and statistical methods used.

Minor comments
1. Overall, the quality of the figures should be improved. Several panels appear to be low resolution, and in some cases plot titles or labels are partially cut off. The authors should revise all figures to ensure that the panels, labels, titles, axes, and legends are clearly readable.
2. The authors should expand the figure legends throughout the manuscript. In particular, each legend should clearly indicate the statistical tests used, the number of animals analyzed, and the number of independent experimental batches or biological replicates included.
3. The movies lack scale bars.
4. Figure S1 is not mentioned in the main text, except in the Methods section. The authors should refer to this figure appropriately in the Results section and ensure that the numbering of supplementary figures follows the order in which they appear in the text.
5. The legend of Figure S2 contains information that appears to belong to the Results section. The authors should move the relevant description into the main text to properly describe the findings shown in the figure.
6. Line 146: please correct the wording to "did not".
7. The authors should better discuss the limitations of the Markov chain approach in the Discussion section, particularly with respect to the assumptions of the method and its ability to capture complex behavioral sequences.
8. The figure titles should be revised to more clearly indicate the main message or meaning of each figure. For example, the title of Figure 3 should more directly describe the biological or analytical result being presented.
9. In Figure 4B, the unit should be added to the y-axis.
10. In Figure 4E, the statistical test used is not specified in the figure legend. In addition, the authors do not describe the B-T phenotype in the main text, although the figure suggests that it is less frequent. This result should be mentioned and discussed.
11. In Figure 4 the panels do not reflect the appereance in the manuscript. At the moment, the appereance is C-E-F-G-D-H.
12. In lines 360-362, the authors refer to Figure S8 and report a significant strain-specific effect in day 1 worms. However, the statement in the text does not appear to accurately reflect the data shown in the figure. The authors should revise this claim.
13. In Figure S8, it is unclear which strain "4022" corresponds to. In addition, the strain JN3038 listed in Table 1 is not mentioned in the manuscript, figures, or figure legends. The authors should clarify these inconsistencies.
14. Lines 377 and 384: references are missing and should be added.

<u>Reviewer 2</u>

Comments for the author

This manuscript presents a quantitative analysis of age-associated locomotor changes in C. elegans by combining eigenworm analysis, time-delay embedding/reconstruction ICA, t-SNE-based behavioral state mapping, and Markov-chain analyses of behavioral transitions. The authors report that aged worms exhibit reduced forward locomotor periodicity, altered transition structure between behavioral states, reduced turn behavior following reversals, and increased inter-individual heterogeneity. The work addresses an important question in aging biology and applies modern behavioral analysis tools to characterize locomotor decline beyond conventional metrics.

© 2026. Published by The Company of Biologists under the terms of the Creative Commons Attribution License (https://creativecommons.org/licenses/by/4.0/).

The study is technically interesting and potentially suitable for publication. However, Novelty and biological significance should be clarified. The manuscript applies several modern behavioral analysis methods (eigenworm analysis, TDE-RICA, t-SNE embedding, and state-transition analysis) to aging-related locomotor decline in C. elegans. While the technical framework is sophisticated, it is not entirely clear what fundamentally new biological insight is obtained beyond the established observation that aged worms exhibit impaired locomotion and altered behavioral organization. The authors should more explicitly articulate how their findings advance the field relative to previous studies of locomotor aging. In particular, the novelty and biological significance of the identified defects in forward-cycle stability and reversal-to-turn transitions should be discussed in greater depth. Are these features merely more sensitive quantitative descriptors of known aging phenotypes, or do they reveal previously unrecognized aspects of nervous system aging? Clarifying this distinction would substantially strengthen the impact of the study.

Several methodological and interpretational issues should also be addressed before publication:
1. Potential circularity and age-dependent embedding construction
The behavioral state map is generated primarily from young animals, and aged animals are subsequently projected into this space. This approach may bias the representation toward young behavioral structure and could exaggerate apparent irregularities in aged animals. The authors should justify this choice more thoroughly and ideally demonstrate that the principal conclusions are robust when embeddings are generated jointly from both groups or using an alternative dimensionality reduction approach.
2. Resampling of young animals
A key step is the interpolation-based slowing of young locomotion to match the undulation frequency of aged worms prior to TDE-RICA. While understandable, this manipulation may alter temporal structure and potentially affect motif extraction and state classification. Additional validation is needed to show that the identified motifs and behavioral states are not artifacts of this preprocessing step.
3. Statistical independence and sample size
Many analyses appear to use events or transitions pooled across animals. It is not always clear whether statistical tests are performed at the level of individual worms or individual events. Because transitions within a worm are not independent observations, the unit of replication should be clearly defined throughout. Reporting the number of animals analyzed in each group would also improve transparency.
4. Interpretation of reversal-turn defects
The reduced probability of turns following reversals is one of the central findings. However, the manuscript occasionally interprets this as impaired reorientation or altered neural organization. These mechanistic conclusions are speculative because no neural measurements are presented. The Discussion should be tempered to distinguish clearly between behavioral observations and proposed neural explanations.
Minor Comments
1. Several references appear to have publication years in the future or incomplete citation (e.g., Gowda et al., 2026). The authors should verify all citations.
2. The manuscript would benefit from a table summarizing sample sizes, number of animals, number of events, and recording durations used in each analysis.
3. The distinction between "turn," "bending," and "C-shape" states should be clarified, preferably with representative images in the main figures rather than relying on supplementary material.
4. The rationale for manually selected thresholds (pause-state definition, angular velocity threshold, max_gap parameter) should be better justified, and sensitivity analyses would strengthen confidence in the conclusions.

Reviewer's Responses to Questions

Experimental quality
Are experiments performed using appropriate methods that will answer the question (or test the hypothesis or support the observations) posed by the authors? Is the right tool used for the job?
If 'No', please indicate reasons in Comments for Author box below.
Reviewer #1:
- Yes

© 2026. Published by The Company of Biologists under the terms of the Creative Commons Attribution License (https://creativecommons.org/licenses/by/4.0/).

Reviewer #2:
- Yes

Were the data analyzed using appropriate statistical tests?
If 'No', please indicate reasons in Comments for Author box below.
Reviewer #1:
- No

Reviewer #2:
- Yes

Reproducibility
Were experiments performed using adequate number of biological replicates?
If 'No', please indicate reasons in Comments for Author box below.
Reviewer #1:
- No

Reviewer #2:
- Yes

Is there sufficient raw data to assess the rigor of the analysis?
If 'No', please indicate reasons in Comments for Author box below.
Reviewer #1:
- Yes

Reviewer #2:
- Yes

Does the methods section provide sufficient detail to permit reproducibility?
If 'No', please indicate reasons in Comments for Author box below.
Reviewer #1:
- No

Reviewer #2:
- Yes

Completeness
Are the manuscript's conclusions supported by the data?
If 'No', please indicate reasons in Comments for Author box below.
Reviewer #1:
- Yes

Reviewer #2:
- Yes

Scholarship
Do the authors cite and discuss the merits of data that would argue for and against their conclusion?
If 'No', please indicate reasons in Comments for Author box below.
Reviewer #1:
- No

Reviewer #2:

© 2026. Published by The Company of Biologists under the terms of the Creative Commons Attribution License (https://creativecommons.org/licenses/by/4.0/).

- Yes

Does the manuscript title & abstract accurately reflect the contents of the manuscript, without hyperbole?
If 'No', please indicate reasons in Comments for Author box below.
Reviewer #1:
- Yes

Reviewer #2:
- Yes

First revision

Author response to reviewers' comments

The reviewers' remarks are underlined and numbered, followed by our one-by-one responses.

Reviewer 1

Issue 1-1
Reviewer 1: Zeng et al. investigate age-associated changes in locomotor behavior using a quantitative behavioral analysis framework in C. elegans. The authors compare young (day 1) and aged (day 9) animals and report a series of aging-associated alterations in locomotor behavior.

Although the manuscript addresses an important biological question and presents a potentially valuable dataset, several aspects of the experimental design and data analysis require substantial clarification. In addition, further validation is needed to support the conclusions and improve the presentation of the data in both the figures and their legends. The manuscript would also benefit from a more balanced discussion of the biological significance of the findings, as well as their limitations and broader implications.
Below are listed the points to address.

Our response:
We are delighted to see the comment that our study addresses an important biological question. Further validation and discussion was added.

Major comments
Issue 1-2
1. The number of worms analyzed in each group, day 1 and day 9, is not reported in the manuscript, figures, figure legends, or Methods section. It is also unclear whether the experiments were performed using a single batch of animals, corresponding to one biological replicate, or whether multiple independent batches were analyzed. This information is essential for evaluating the robustness of the results and the appropriateness of the statistical analyses. The authors should clearly report the number of animals, the number of independent biological replicates, and how these were incorporated into the statistical analysis. Ideally, the experiments should be performed across multiple independent batches, or alternatively the authors should provide a power analysis justifying the sample size used.

Our response:
The sample size was 16 animals in each group, which is relatively modest compared with some traditional behavioral studies. Nevertheless, each animal was recorded for 5 min at 5 fps, resulting in a sufficient number of time frames for quantifying behavioral dynamics and state transitions (Figure 3). We agree that batch effects can influence behavioral analyses. To reduce this concern, animals from multiple batches were included for both the TOY21 and N2 strain analyses. This information has now been added to the Methods section.

© 2026. Published by The Company of Biologists under the terms of the Creative Commons Attribution License (https://creativecommons.org/licenses/by/4.0/).

Issue 1-3

2. The information regarding the strain used in the study requires clarification. In particular, the rationale for using the TOY21 strain should be explained, including whether this strain carries genetic modifications that could influence locomotor behavior or aging-related phenotypes. To strengthen the generality of the conclusions, the key findings should also be validated in a standard wild-type strain.

Our response:

The genetic background of the TOY21 strain was summarized in Table 1. We originally planned to perform whole-brain imaging in freely moving worms while simultaneously recording their locomotion, which would help us better understand the relationship between aging and neuroscience. However, neuron tracking in freely moving worms is challenging. Moreover, aged worms have a greater body diameter, making it even more difficult to capture neurons located in the deeper half of the body, due to the optical limitation. Therefore, we were unable to obtain reliable neural activity data in the present study.

We added this information to the Discussion section. In addition, analysis using the wild-type strain has been added to the Supplementary Information (Figs. S12-S15).

Issue 1-4

3. In Figure 2A, the authors present the analytical pipeline. However, the schematic representation of the different analytical steps is relatively complex and difficult to follow. The authors should provide additional clarification in the figure legend and/or simplify the schematic. Alternatively, this panel may be more suitable as a dedicated supplementary figure, with the main text focusing on the key conceptual steps of the analysis.

Our response:

We added further explanation in the legend for this figure and moved it to the Supplementary Information.

Issue 1-5

4. In Figure 2C, the authors present a clustering analysis based on the generation of a density map in a two-dimensional projection of the multidimensional t-SNE space, followed by watershed-based cluster identification. Two major concerns arise from this approach. First, the method is not sufficiently justified and appears unconventional for this type of analysis, as clustering is performed on a two-dimensional projection rather than in the original multidimensional feature space. More standard unsupervised clustering approaches, such as k-nearest-neighbor-based clustering, Louvain/Leiden clustering, or other methods applied before or independently of dimensionality reduction, would be more appropriate and less dependent on the distortions introduced by t-SNE visualization.

Second, the cluster boundaries shown in the figure do not appear to match the density distribution of the data particularly well and do not seem to capture the organization of the young and aged worm datasets in a convincing way. The authors should therefore repeat or validate the clustering analysis using dimensionality reduction methods such as t-SNE or UMAP coupled with clustering approaches that are not based solely on two-dimensional projections. This would help determine whether the identified behavioral clusters are robust and not an artefact of the visualization method.

Our response:

We agree that clustering based solely on a two-dimensional t-SNE density map may be influenced by the dimensionality reduction and by the parameters used for density estimation and watershed segmentation. Our analysis followed the behavioral mapping framework introduced by Berman et al, 2014 on *Drosophila* and Liu et al, 2018, in which behavioral states are identified from a low-dimensional t-SNE representation of postural dynamics. Nevertheless, we agree that additional validation is necessary to ensure that the identified behavioral clusters are not simply artefacts of the two-dimensional visualization. We added further analysis to the main text.

Issue 1-6

© 2026. Published by The Company of Biologists under the terms of the Creative Commons Attribution License (https://creativecommons.org/licenses/by/4.0/).

5. In Figure 2C, the authors indicate the presence of 11 clusters. However, in Figure 2D the clusters are labeled from 0 to 11, which corresponds to 12 clusters. In addition, "cluster 12" is mentioned in the text at line 162. The authors should carefully revise this section and ensure consistency among the text, figures, labels, and analysis.

Our response:
The explanation of cluster 0 and cluster 12 was provided in line 158-162 of the original manuscript. To avoid confusion and clarify the meanings of clusters 0 and 12, we changed the representative samples shown in Figure 2C (This is now Figure 2B, because the original Figure 2A has been moved to the Supplementary Information in response to Issue 1-4).

Issue 1-7
6. In Figures 2E-G, it is unclear how many worms were included in the analysis. The figure legends do not provide sufficient information to understand the sample size, the number of biological replicates, or how the data were aggregated. The authors should clarify these points in the legends and Methods section.

Our response:
We added this information in the legends and Methods section.

Issue 1-8
7. In Figures 3A-B, the subtraction matrix appears to provide only a descriptive comparison between groups, without statistical testing to support the reported differences. The authors should include appropriate statistical analyses to assess whether the observed changes in transition probabilities are significant. For example, they could apply a chi-square test, or calculate transition probabilities for each individual worm and then perform statistical testing across biological replicates. This would provide stronger support for the conclusions drawn from these data.

Our response:
We calculated transition probabilities for each worm and then performed statistical tests. This information is shown in the revised Figure 3A and B.

Issue 1-9
8. In Figure 5, panels A and B appear to be switched. The descriptions in the text and figure legend do not match the corresponding panels. The authors should correct the figure, legend, and text to ensure consistency.

Our response:
We changed the order of these two figures.

Issue 1-10
9. In Figure 5, it is unclear whether the calculations are based on the t-SNE analysis shown in Figure 2. This information is missing from both the main text and the figure legend. The authors should clearly explain how these calculations were performed and whether they derive from the same dimensionality-reduction space used in Figure 2.

Our response:
The figures in the original manuscript were generated from a dataset that combined with body-axis angular velocity. In this revised manuscript, we replaced this with the dataset obtained directly from t-SNE analysis described in Figure 2. We added this information in the Methods section.

Issue 1-11
10. In the Discussion section, the authors should provide appropriate references for all major claims, particularly in the section comparing worm locomotor aging with human aging. In addition, lines 405-408 appear to contain repetitive statements and should be revised for clarity. The Discussion would also benefit from a more explicit consideration of the limitations of the current study, including limitations related to the experimental design, sample size, behavioral classification approach, and statistical methods used.

Our response:

© 2026. Published by The Company of Biologists under the terms of the Creative Commons Attribution License (https://creativecommons.org/licenses/by/4.0/).

We have added references and discussed the limitations of this work in the discussion section.

Issue 1-12
Minor comments
1. Overall, the quality of the figures should be improved. Several panels appear to be low resolution, and in some cases plot titles or labels are partially cut off. The authors should revise all figures to ensure that the panels, labels, titles, axes, and legends are clearly readable.

Our response:
We improved the quality of all figures.

Issue 1-13
2. The authors should expand the figure legends throughout the manuscript. In particular, each legend should clearly indicate the statistical tests used, the number of animals analyzed, and the number of independent experimental batches or biological replicates included.

Our response:
We revised the figure legends to provide more detailed explanations and clarified the relevant information as suggested.

Issue 1-14
3. The movies lack scale bars.

Our response:
We added the scale bars to the movies.

Issue 1-15
4. Figure S1 is not mentioned in the main text, except in the Methods section. The authors should refer to this figure appropriately in the Results section and ensure that the numbering of supplementary figures follows the order in which they appear in the text.

Our response:
We added some descriptions in lines 86-87 and 142-143.

Issue 1-16
5. The legend of Figure S2 contains information that appears to belong to the Results section. The authors should move the relevant description into the main text to properly describe the findings shown in the figure.

Our response:
This information as in the caption for Fig S2 has already been included to the Results section in the original manuscript (Line 91)

Issue 1-17
6. Line 146: please correct the wording to "did not".

Our response:
We have corrected the word.

Issue 1-18
7. The authors should better discuss the limitations of the Markov chain approach in the Discussion section, particularly with respect to the assumptions of the method and its ability to capture complex behavioral sequences.

Our response:
We added these limitations in the last paragraph of the Discussion section.

Issue 1-19

© 2026. Published by The Company of Biologists under the terms of the Creative Commons Attribution License (https://creativecommons.org/licenses/by/4.0/).

8. The figure titles should be revised to more clearly indicate the main message or meaning of each figure. For example, the title of Figure 3 should more directly describe the biological or analytical result being presented.

Our response:
We revised all figures and added the corresponding statistical analyses to the relevant figures.

Issue 1-20
9. In Figure 4B, the unit should be added to the y-axis.

Our response:
The y-axis in Figure 4B indicates the empirical cumulative probability and therefore has no unit. We revised the title and x-axis of this figure to make it clearer.

Issue 1-21
10. In Figure 4E, the statistical test used is not specified in the figure legend. In addition, the authors do not describe the B-T phenotype in the main text, although the figure suggests that it is less frequent. This result should be mentioned and discussed.

Our response:
We added the description of the statistical test in the legend.
B-T behavior was described in line 241 and lines 248-250.

Issue 1-22
11. In Figure 4 the panels do not reflect the appearance in the manuscript. At the moment, the appearance is C-E-F-G-D-H.

Our response:
We revised the order of the panel.

Issue 1-23
12. In lines 360-362, the authors refer to Figure S8 and report a significant strain-specific effect in day 1 worms. However, the statement in the text does not appear to accurately reflect the data shown in the figure. The authors should revise this claim.

Our response:
To better demonstrate the robustness of our study, we applied the same pipeline to analyze the wild-type strain and added these results to the Supplementary Information. Accordingly, the original Figure S8 has been deleted.

Because both the JN3038 and TOY21 strains were generated for whole-brain imaging and carry multiple fluorescent markers for neuronal annotation and activity imaging, they may not be ideal references for behavioral analysis alone. Therefore, in the revised manuscript, we used the wild-type strain instead of the JN3038 strain for the supplementary analysis.

Issue 1-24
13. In Figure S8, it is unclear which strain "4022" corresponds to. In addition, the strain JN3038 listed in Table 1 is not mentioned in the manuscript, figures, or figure legends. The authors should clarify these inconsistencies.

Our response:
Similar to our response to Issues 1-3, Issues 1-23, we revised the analysis to include the wild-type strain. Accordingly, we deleted Figure S8 and removed the analyses related to the “4022” (JN3038) strain. In the revised manuscript, only the wild-type strain is used for this analysis.

Issue 1-25
14. Lines 377 and 384: references are missing and should be added.

Our response:
We have added the references.

© 2026. Published by The Company of Biologists under the terms of the Creative Commons Attribution License (https://creativecommons.org/licenses/by/4.0/).

Reviewer 2:
Issue 2-1
This manuscript presents a quantitative analysis of age-associated locomotor changes in C. elegans by combining eigenworm analysis, time-delay embedding/reconstruction ICA, t-SNE-based behavioral state mapping, and Markov-chain analyses of behavioral transitions. The authors report that aged worms exhibit reduced forward locomotor periodicity, altered transition structure between behavioral states, reduced turn behavior following reversals, and increased inter-individual heterogeneity. The work addresses an important question in aging biology and applies modern behavioral analysis tools to characterize locomotor decline beyond conventional metrics.

The study is technically interesting and potentially suitable for publication. However, Novelty and biological significance should be clarified. The manuscript applies several modern behavioral analysis methods (eigenworm analysis, TDE-RICA, t-SNE embedding, and state-transition analysis) to aging-related locomotor decline in C. elegans. While the technical framework is sophisticated, it is not entirely clear what fundamentally new biological insight is obtained beyond the established observation that aged worms exhibit impaired locomotion and altered behavioral organization. The authors should more explicitly articulate how their findings advance the field relative to previous studies of locomotor aging. In particular, the novelty and biological significance of the identified defects in forward-cycle stability and reversal-to-turn transitions should be discussed in greater depth. Are these features merely more sensitive quantitative descriptors of known aging phenotypes, or do they reveal previously unrecognized aspects of nervous system aging? Clarifying this distinction would substantially strengthen the impact of the study.

Our response:
We are delighted to see the comment that our study addresses an important biological question. We added some discussion in the Discussion section.

Issue 2-2
1. Potential circularity and age-dependent embedding construction
The behavioral state map is generated primarily from young animals, and aged animals are subsequently projected into this space. This approach may bias the representation toward young behavioral structure and could exaggerate apparent irregularities in aged animals. The authors should justify this choice more thoroughly and ideally demonstrate that the principal conclusions are robust when embeddings are generated jointly from both groups or using an alternative dimensionality reduction approach.

Our response:
We agree that it is more common to generate a t-SNE map using the combined young and aged dataset. We had actually tried this approach before, however, we found that the young and aged group occupied almost distinct regions in the map, which made direct comparison difficult (Fig. S6). Therefore, in this study, we generated the t-SNE map using the young dataset and projected the aged animals into this space.

Issue 2-3
2. Resampling of young animals
A key step is the interpolation-based slowing of young locomotion to match the undulation frequency of aged worms prior to TDE-RICA. While understandable, this manipulation may alter temporal structure and potentially affect motif extraction and state classification. Additional validation is needed to show that the identified motifs and behavioral states are not artifacts of this preprocessing step.

Our response:
We appreciate the reviewer pointing out the potential artifacts that could be introduced by matching the undulation frequency.
Our original goal was to identify the qualitative differences in motifs that represent distinct behavioral patterns between the young and aged groups, rather than a simple decline in the locomotion cycle speed during aging. Therefore, we normalized the undulation frequencies of the young and aged groups before performing the TDE-RICA analysis to decouple velocity from posture.

© 2026. Published by The Company of Biologists under the terms of the Creative Commons Attribution License (https://creativecommons.org/licenses/by/4.0/).

To address the concern that this interpolation might distort the motif extraction and state classification, we processed both the original and resampled young datasets through the exact same analysis pipeline. As a result, we found that resampling did not substantially alter the extracted motifs, their occurrence frequencies, or the resulting behavioral clusters (Fig. S4). Therefore, we are confident that resampling the young dataset is a reasonable preprocessing step that does not compromise or artificially affect conclusions presented in this manuscript.

Issue 2-4
3. Statistical independence and sample size
Many analyses appear to use events or transitions pooled across animals. It is not always clear whether statistical tests are performed at the level of individual worms or individual events. Because transitions within a worm are not independent observations, the unit of replication should be clearly defined throughout. Reporting the number of animals analyzed in each group would also improve transparency.

Our response:
Similar to our response to Issue 1-13, the statistical analysis was performed at the level of individual worms. We added this information to the related figure legends. We also added the description of the sample size in the Methods section.

Issue 2-5
4. Interpretation of reversal-turn defects
The reduced probability of turns following reversals is one of the central findings. However, the manuscript occasionally interprets this as impaired reorientation or altered neural organization. These mechanistic conclusions are speculative because no neural measurements are presented. The Discussion should be tempered to distinguish clearly between behavioral observations and proposed neural explanations.

Our response:
We carefully checked the manuscript and revised the relevant descriptions to avoid implying a direct link between neural activity and behavioral defects. In the revised manuscript, we mentioned neural activity as a future perspective.

Issue 2-6
Minor Comments
1. Several references appear to have publication years in the future or incomplete citation (e.g., Gowda et al., 2026). The authors should verify all citations.

Our response:
We have verified all the citations.

Issue 2-7
2. The manuscript would benefit from a table summarizing sample sizes, number of animals, number of events, and recording durations used in each analysis.

Our response:
1) The information of the sample size was added to the Methods section.
2) The information of the number of events and other relevant parameters were provided as an Excel file in the Supplementary Information.
3) The information of the recording duration was described in the Methods section.

Issue 2-8
3. The distinction between "turn," "bending," and "C-shape" states should be clarified, preferably with representative images in the main figures rather than relying on supplementary material.

Our response:
We added representative worm-shape images for these three clusters to the Results section. To describe the body shapes more accurately and avoid confusion, we changed “C-shape” to “U-shape.”

© 2026. Published by The Company of Biologists under the terms of the Creative Commons Attribution License (https://creativecommons.org/licenses/by/4.0/).

Issue 2-9
4. The rationale for manually selected thresholds (pause-state definition, angular velocity threshold, max_gap parameter) should be better justified, and sensitivity analyses would strengthen confidence in the conclusions.

Our response:
We added some description and reference to support the validity of these manually selected thresholds.

Second decision letter

MS ID#: bio.062711R1

MS Title: Aging related behavioral defects in C. elegans

Authors: Jingxuan Zeng, Chung-Kuan Chen, Yuichi Iino and Yu Toyoshima

I am happy to tell you that your manuscript has been accepted for publication in Biology Open, pending our standard publication integrity checks. It was accepted on 23rd July 2026.

© 2026. Published by The Company of Biologists under the terms of the Creative Commons Attribution License (https://creativecommons.org/licenses/by/4.0/).
